# Sex Differences in Cardiovascular Disease Outcomes After Traumatic Brain Injury

**DOI:** 10.1016/j.jacadv.2026.102658

**Published:** 2026-03-18

**Authors:** John E. Balke, Ian J. Stewart, Megan E. Amuan, Jeffrey T. Howard, Jill E. Brown, Eamonn Kennedy, Mark Haigney, Mary Jo Pugh

**Affiliations:** a Department of Medicine, Uniformed Services University, Bethesda, Maryland, USA; b Military Cardiovascular Outcomes Research Program, Bethesda, Maryland, USA; c Informatics, Decision-Enhancement, and Analytic Sciences Center of Innovation, VA Salt Lake City Health Care System, Salt Lake City, Utah, USA; d Division of Epidemiology, Department of Internal Medicine, University of Utah School of Medicine, Salt Lake City, Utah, USA; e Department of Public Health, University of Texas at San Antonio, San Antonio, Texas, USA; f Department of Gynecologic Surgery and Obstetrics, Uniformed Services University, Bethesda, Maryland, USA

**Keywords:** coronary artery disease, peripheral artery disease, stroke, traumatic brain injury, woman’s health

## Abstract

**Background:**

We previously demonstrated that traumatic brain injury (TBI) is associated with an increased risk of cardiovascular disease (CVD) in a large cohort of post-9/11 service members and veterans.

**Objectives:**

As emerging evidence indicates that men and women have different outcomes after TBI, we sought to describe CVD risk after TBI stratified by sex.

**Methods:**

Data were obtained from the Long-term Impact of Military-Relevant Brain Injury Consortium-Chronic Effects of Neurotrauma Consortium Phenotype study. The cohort was divided into 2 subgroups: men and women. The primary outcome of interest was CVD defined as a composite of coronary artery disease, stroke, peripheral artery disease, and CVD mortality defined by International Classification of Diseases diagnosis codes. We performed Fine-Gray competing risks analyses to determine the association of TBI severity with subsequent CVD.

**Results:**

The study cohort consisted of N = 1,277,430 men and N = 282,498 women, with TBI prevalence of 20.8% and 12.7%, respectively. Men had a higher prevalence of CVD risk factors, and CVD was more prevalent in men (3.1%) compared to women (1.9%) (*P* < 0.001). In fully adjusted models, HRs and 95% CIs for CVD risk were higher in women than in men for all levels of TBI: HR for mild TBI in women: 2.09 (2.02-2.16) vs men: 1.62 (1.60-1.64); HR for moderate/severe TBI in women: 3.45 (3.34-3.56) vs men: 2.65 (2.62-2.69); HR for penetrating TBI among women: 5.61 (5.44-5.78) vs men 4.22 (4.17-4.27).

**Conclusions:**

We found that while CVD is less common in women, TBI is associated with a larger risk for subsequent CVD in women. Future work is needed to determine the etiology of this association to improve long-term care given the increase in female service members in combat.

The injuries service members obtained during the wars in Iraq and Afghanistan have continued health effects with a significant ongoing cost. Of the 4.5 million people who have served in the U.S. military since the attacks on September 11, 2001,^1^ it is estimated that up to 20% have suffered a traumatic brain injury (TBI) in the course of that service.^2^ TBI is a risk factor in the development of dementia and has been associated with elevated risk of other chronic diseases, ranging from sleep and psychiatric disorders, to genitourinary and neuroendocrine dysfunction.^3^ Additionally, evidence suggests that TBI is linked to an increased risk of cardiovascular disease (CVD), encompassing stroke, coronary artery disease (CAD), and peripheral artery disease, that persists across all TBI severity levels to include mild, moderate/severe, and penetrating injuries.^4^ The relationship between TBI and CVD risk has been demonstrated in cohorts predominantly composed of younger populations, underscoring that TBI may contribute to CVD development even among individuals traditionally considered low risk.^4^

A previous study showed that from 2002 to 2016, 32.4% (N = 29,735) of injured service members reaching medical facilities in Iraq and Afghanistan had a TBI. In this cohort, TBI was more common in males (32.6%) compared to females (25.3%).^5^ However, following the Department of Defense’s 2013 decision to rescind the direct ground combat exclusion rule for female service members,^6^ the proportion of female military personnel experiencing TBI is anticipated to rise. Research indicates that women may suffer worse outcomes after TBI than men.^7^ A recent study examining the relationship between TBI and subsequent atrial fibrillation/atrial flutter (AF/AFL) demonstrated that females, who typically exhibit a lower baseline risk of AF/AFL, had a higher risk of subsequent AF/AFL following TBI compared to their male counterparts.^8^ Given the established association between TBI and elevated CVD risk and sex-specific differences in AF/AFL incidence post-TBI, we hypothesize that sex may also modulate the relationship between TBI and CVD. Specifically, we anticipated that female service members will exhibit distinct patterns of CVD development following TBI.

## Methods

The methods for cohort derivation and covariate definitions have been previously published.^4^ Briefly, the protocol was reviewed and approved by the University of Utah Institutional Review Board in accordance with all relevant federal regulations. Given the retrospective nature of the work, the Institutional Review Board granted a waiver of informed consent. Data were obtained from the Long-term Impact of Military-Relevant Brain Injury Consortium (LIMBIC) Phenotype study. The LIMBIC Phenotype study obtained data from various databases, including the U.S. Department of Veterans Affairs (VA) and Department of Defense (DoD) Identity Repository (VADIR), VA Corporate Data Warehouse, DoD and VA Infrastructure for Clinical Intelligence, Theater Data Management Store, DoD Trauma Registry, and the National Death Index (NDI). To be included in the LIMBIC cohort, service members and veterans were required to have at least 3 years of health care within the DoD system and, for those entering the VA, a minimum of 2 years of VA health care between FY00 and FY19 (October 1, 1999, and September 30, 2019). To ensure sufficient follow-up time for participants to develop the outcome, subjects were excluded from this analysis if they had an index date on or after October 1, 2016. Subjects were also excluded if they had a single outpatient CVD diagnosis (unless associated with a procedure or CVD death), a prior CVD diagnosis before the index date, death before the index date (in the non-TBI cohort), were under the age of 17 at the index date, had an index date before DoD care or after the last health care utilization, or had an ambiguous TBI status. The cohort was then divided into 2 groups on the basis of sex. The study complied with the Strengthening the Reporting of Observational Studies in Epidemiology guidelines.

Demographic covariates extracted from VADIR included age, sex, race and ethnicity, education, military component, rank, deployment history, and combat exposure. Service branch information was primarily obtained from Corporate Data Warehouse, with VADIR used as a secondary source if necessary. Smoking status was determined by International Classification of Diseases-9th and -10th Clinical Modification (ICD-9-CM and ICD-10-CM) codes, prescription for smoking cessation medications, or a positive clinical screen. Mortality data were obtained from the VA vital status files and the NDI. Additional covariates, such as hypertension, diabetes, obesity, kidney disease, hyperlipidemia, obstructive sleep apnea, insomnia, depression, posttraumatic stress disorder (PTSD), anxiety, and substance use disorder, were identified through ICD-9-CM and ICD-10-CM diagnosis codes (Supplemental Table 1).

The primary variable of interest was TBI defined in a hierarchical manner as previously described.^4^ Briefly, data on traumatic injury were taken from the DoD Trauma Registry and included both injury codes and the Glasgow Coma Scale. TBI was also defined by self-report, with mild TBI defined as loss of consciousness for ≤30 minutes or alteration of consciousness/amnesia for <24 hours and moderate to severe TBI defined as loss of consciousness for >30 minutes or alteration of consciousness/amnesia for ≥24 hours. Finally, TBI was defined by ICD-9-CM and ICD-10-CM codes from DoD and VA medical records. The primary outcome of interest was CVD, which was defined as a composite outcome inclusive of CAD, stroke, peripheral artery disease (PAD), and CVD mortality defined by ICD-9-CM and ICD-10-CM diagnosis codes from the medical record. For a CVD diagnosis, subjects were required to have either 1 inpatient visit or 2 outpatient visits with a defined CVD diagnostic code spaced at least 7 days apart (with the exception of CVD mortality).

We first examined the fully adjusted model from our prior study,^4^ incorporating an interaction term by sex. Analysis of the interaction between sex and TBI indicate that the HRs were 45%, 44%, and 62% greater in females for mild (HR: 1.45; 95% CI: 1.40-1.50; *P* < 0.001), moderate/severe (HR: 1.44; 95% CI: 1.40-1.49; *P* < 0.001), and penetrating (HR: 1.62; 95% CI: 1.56-1.67; *P* < 0.001) TBI, respectively (Supplemental Table 2). Given the significance of this interaction, subgroup analyses were conducted for men and women to separately assess TBI-CVD relationships by group. Standard descriptive statistics were used to describe the differences between men and women. The characteristics of service members with and without TBI were also compared within each group. The association between TBI and subsequent CVD was evaluated using Fine-Gray competing risk models^9^ stratified by sex. For veterans diagnosed with TBI, the index date corresponded to the date of the first TBI diagnosis. For veterans without TBI, index dates were simulated based on the distribution of actual index dates within respective age groups.^10^ HRs and 95% CIs were calculated for each TBI severity category using bivariate models and multivariable models. We limited the covariate adjustment to comorbidities with a strong causal association with CVD. The multivariable models adjusted for birth year, race and ethnicity, education, service branch, component, rank, deployment history, smoking history, substance use disorder, obesity, depression, anxiety, insomnia, PTSD, hyperlipidemia, hypertension, kidney disease, diabetes, and obstructive sleep apnea. Model assumptions were tested by examining interactions between log(time) and TBI. This demonstrated the hazard associated with each TBI severity category decreases over time. Visual inspection of the cumulative incidence curves suggested that departures from proportionality are modest and do not materially affect conclusions; we therefore summarize effects using average HRs over time for interpretability.

Cumulative incidence functions (CIFs) were plotted separately for men and women veterans to visualize sex-specific differences in cumulative risk. Propensity score weighting across TBI groups was used in the estimation of CIFs, which were developed using the SAS PHREG (SAS Institute) procedure by fitting Fine-Gray hazard models across groups. In a secondary analysis again stratified by sex, the impact of TBI on each of the individual components of the primary endpoint was evaluated (CAD, stroke, PAD, and CVD death).

## Results

**Figure 1:**
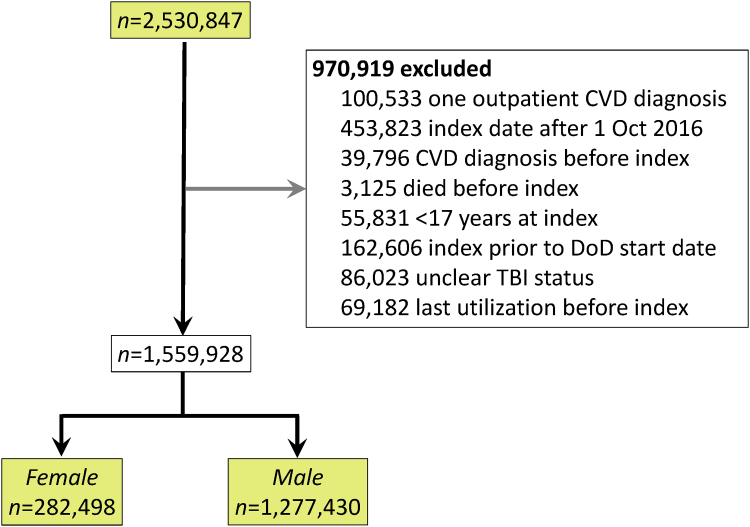
**Cohort Design** Consort diagram showing the development of the study cohort. CVD = cardiovascular disease; DoD = Department of Defense; TBI = traumatic brain injury.

The summary of cohort derivation and results are shown in the Central Illustration. The LIMBIC-Chronic Effects of Neurotrauma Consortium (CENC) Phenotype study initially included a total of 2,530,847 veterans. After applying exclusion criteria, 970,919 veterans were removed from the cohort, resulting in a final cohort size of 1,559,928 veterans for analysis (Figure 1). Of the 1,559,928 veterans in the study cohort, 81.9% (1,277,430) were men and 18.1% (282,498) were women. Compared to men, women were slightly younger (median age 28 vs 29 years), less likely to have TBI (12.7% vs 20.8%), more likely to have a post-high school education (36.0% vs 29.1%), and less likely to have deployed and seen combat (48.6% vs 69%) (Table 1). Men were more likely to have CVD risk factors to include smoking (44.9% vs 34.2%), hyperlipidemia (15.9% vs 9.5%), kidney disease (0.5% vs 0.2%), hypertension (12.0% vs 7.7%), and diabetes (2.0% vs 1.5%). CVD was significantly more common in men (3.1%) than in women (1.9%).

**Central Illustration:**
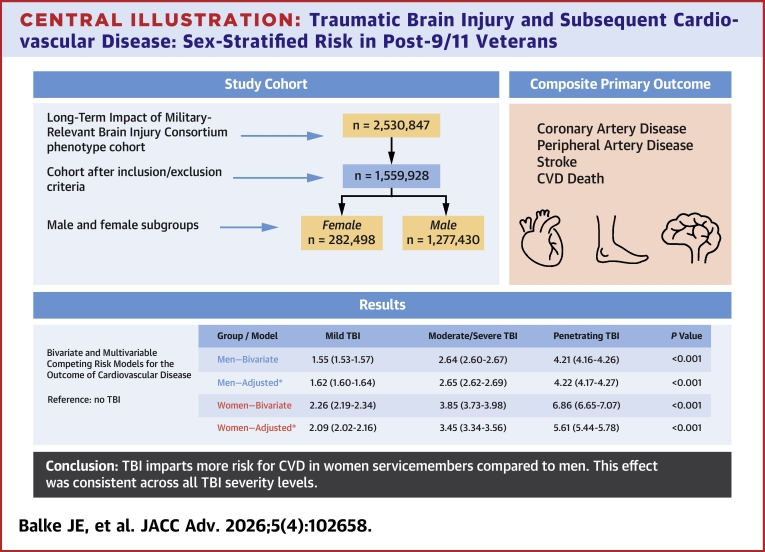
**Traumatic Brain Injury and Subsequent Cardiovascular Disease: Sex-Stratified Risk in Post-9/11 Veterans** Adjusted for demographics, military, and clinical covariates as in manuscript. Abbreviations as in Figure 1.

**Table 1:** Characteristics of the Study Cohort

	Total Cohort (N = 1,559,928)	Male (n = 1,277,430, 81.9%)	Female (n = 282,498, 18.1%)	*P* Value
Age, y (median, IQR)	29 (24-38)	29 (24-39)	28 (24-36)	<0.001
Traumatic brain injury	301,169 (19.3)	265,271 (20.8)	35,898 (12.7)	<0.001
Race and ethnicity				<0.001
Asian/Pacific Islander	96,560 (6.2)	80,098 (6.3)	16,462 (5.8)	
Hispanic	149,622 (9.6)	121,734 (9.5)	27,888 (9.9)	
Hispanic Black	8,408 (0.5)	6,201 (0.5)	2,207 (0.8)	
Native American	26,752 (1.7)	20,663 (1.6)	6,089 (2.2)	
Non-Hispanic Black	267,992 (17.2)	193,042 (15.1)	74,950 (26.5)	
Non-Hispanic White	983,977 (63.1)	838,126 (65.1)	145,851 (51.6)	
Unknown	26,617 (1.7)	17,566 (1.4)	9,051 (3.2)	
Education				<0.001
Less than high school	22,218 (1.4)	18,375 (1.4)	3,843 (1.4)	
High school	1,047,158 (67.1)	874,881 (68.5)	172,277 (61.0)	
High school	472,976 (30.3)	371,333 (29.1)	101,643 (36.0)	
Unknown	17,576 (1.1)	12,841 (1.0)	4,735 (1.7)	
Service branch				<0.001
Army	708,174 (45.4)	579,694 (45.4)	128,480 (45.5)	
Air force	306,481 (19.7)	234,107 (18.3)	72,374 (25.6)	
Marines	234,879 (15.1)	216,284 (16.9)	18,595 (6.6)	
Navy/coast guard	308,755 (19.8)	246,451 (19.3)	62,304 (22.1)	
Other	1,639 (0.1)	894 (0.1)	745 (0.3)	
Component				0.511
Guard/reserve	759,273 (48.7)	621,613 (48.7)	137,660 (48.7)	
Active	800,655 (51.3)	655,817 (51.3)	144,838 (51.3)	
Rank				<0.001
Officer/warrant	215,083 (13.8)	172,792 (13.5)	42,291 (15.0)	
Enlisted	1,344,845 (86.2)	1,104,638 (86.5)	240,207 (85.0)	
Deployment history				<0.001
+Combat and deploy	1,018,676 (65.3)	881,257 (69.0)	137,419 (48.6)	
+Combat/-deploy	76,698 (4.9)	65,177 (5.1)	11,521 (4.1)	
-Combat/+deploy	78,778 (5.1)	63,667 (5.0)	15,111 (5.4)	
-Combat or deploy	385,776 (24.7)	267,329 (20.9)	118,447 (41.9)	
Smoking history	670,521 (43.0)	574,043 (44.9)	96,478 (34.2)	<0.001
Substance use disorder	178,371 (11.4)	151,244 (11.8)	27,127 (9.6)	<0.001
Obesity	195,484 (12.5)	147,236 (11.5)	48,248 (17.1)	<0.001
Obstructive sleep apnea	89,409 (5.7)	82,674 (6.5)	6,735 (2.4)	<0.001
Insomnia	154,655 (9.9)	120,836 (9.5)	33,819 (12.0)	<0.001
Post-traumatic stress disorder	164,773 (10.6)	139,244 (10.9)	25,529 (9.0)	<0.001
Depression	232,176 (14.9)	166,181 (13.0)	65,995 (23.4)	<0.001
Anxiety	179,777 (11.5)	131,850 (10.3)	47,927 (17.0)	<0.001
Hyperlipidemia	230,412 (14.8)	203,570 (15.9)	26,842 (9.5)	<0.001
Kidney disease	6,329 (0.4)	5,696 (0.5)	633 (0.2)	<0.001
Hypertension	174,914 (11.2)	153,067 (12.0)	21,847 (7.7)	<0.001
Diabetes	29,869 (1.9)	25,527 (2.0)	4,342 (1.5)	<0.001
Cardiovascular disease	44,763 (2.9)	39,350 (3.1)	5,413 (1.9)	<0.001

The characteristics of the women veterans in the study cohort stratified by TBI level are shown in Table 2. Significant differences were observed across age groups, with the 25 to 34 age range representing the largest proportion in each category. Racial, ethnic, and education level distributions also varied significantly by TBI status (*P* < 0.001). Service branch distributions reflected significant differences in TBI history (*P* < 0.001). Across service branches, Army veterans had the highest rates of TBI. Deployment history showed a clear association with TBI severity (*P* < 0.001). Mental health conditions, such as PTSD, depression, and anxiety, were more prevalent among those with moderate-to-severe TBI (31.8%, 42.7%, 34.7%, respectively) compared to all other levels of TBI severity (*P* < 0.001). Cardiovascular risk factors were increased in the moderate/severe and penetrating TBI groups vs the mild TBI and no-TBI groups.

**Table 2:** Characteristics of Females in the Study Cohort

	No TBI N = 246,600 (87.3%)	Mild TBI N = 31,147 (11.0%)	Moderate or Severe TBI N = 3,912 (1.4%)	Penetrating TBI N = 839 (0.3%)	*P* Value
Age, y (median, IQR)	28 (24-36)	26 (22-33)	28 (24-36)	29 (23-38)	<0.001
Race and ethnicity					<0.001
Asian/Pacific Islander	14,217 (5.8)	1,937 (6.2)	262 (6.7)	46 (5.5)	
Hispanic	24,278 (9.9)	3,113 (10.0)	406 (10.4)	91 (10.9)	
Hispanic Black	1,960 (0.8)	217 (0.7)	21 (0.5)	≤10 (≤1.2)	
Native American	5,132 (2.1)	802 (2.6)	130 (3.3)	25 (3.0)	
Non-Hispanic Black	66,351 (26.9)	7,564 (24.3)	831 (21.2)	204 (24.3)	
Non-Hispanic White	126,542 (51.3)	16,677 (53.5)	2,191 (56.0)	441 (52.6)	
Unknown	8,120 (3.3)	837 (2.7)	71 (1.8)	23 (2.7)	
Education					<0.001
Less than high school	3,383 (1.4)	403 (1.3)	49 (1.3)	≤10 (≤1.2)	
High school	148,766 (60.3)	20,516 (65.9)	2,522 (64.5)	473 (56.4)	
>High school	90,225 (36.3)	9,778 (31.4)	1,300 (33.2)	340 (40.5)	
Unknown	4,226 (1.7)	450 (1.4)	41 (1.1)	18 (2.2)	
Service branch					<0.001
Army	109,124 (44.3)	16,717 (53.7)	2,153 (55.0)	486 (57.9)	
Air force	65,059 (26.4)	6,390 (20.5)	769 (19.7)	156 (18.6)	
Marines	15,948 (6.5)	2,286 (7.3)	297 (7.6)	64 (7.6)	
Navy/coast guard	55,788 (22.6)	5,704 (18.3)	683 (17.5)	129 (15.4)	
Other	681 (0.3)	50 (0.2)	≤10 (≤0.3)	≤10 (≤1.2)	
Component					<0.001
Guard/reserve	122,055 (49.5)	13,705 (44.0)	1,628 (41.6)	272 (32.4)	
Active	124,545 (50.5)	17,442 (56.0)	2,284 (58.4)	567 (67.6)	
Rank					<0.001
Officer/warrant	38,168 (15.5)	3,493 (11.2)	483 (12.4)	147 (17.5)	
Enlisted	208,432 (84.3)	27,654 (88.8)	3,429 (87.7)	692 (82.5)	
Deployment history					<0.001
+Combat and deploy	117,621 (47.7)	17,032 (54.7)	2,326 (59.5)	440 (52.4)	
+Combat/-deploy	10,455 (4.2)	914 (2.9)	120 (3.1)	32 (3.8)	
-Combat/+deploy	13,285 (5.4)	1,624 (5.2)	168 (4.3)	34 (4.1)	
-Combat or deploy	105,239 (42.7)	11,577 (37.2)	1,298 (33.2)	333 (39.7)	
Smoking history	80,187 (32.5)	14,021 (45.0)	1,870 (47.8)	400 (47.7)	<0.001
Substance use disorder	22,302 (9.0)	4,002 (12.9)	689 (17.6)	134 (16.0)	<0.001
Obesity	41,949 (17.0)	5,388 (17.3)	743 (19.0)	168 (20.0)	<0.001
Obstructive sleep apnea	5,573 (2.3)	922 (3.0)	192 (4.9)	48 (5.7)	<0.001
Insomnia	26,531 (10.8)	5,960 (19.1)	1,063 (27.2)	265 (31.6)	<0.001
Post-traumatic stress disorder	18,405 (7.5)	5,689 (18.3)	1,242 (31.8)	193 (23.0)	<0.001
Depression	53,886 (21.9)	10,120 (32.5)	1,670 (42.7)	319 (38.0)	<0.001
Anxiety	38,464 (15.6)	7,826 (25.1)	1,359 (34.7)	278 (33.1)	<0.001
Hyperlipidemia	23,687 (9.6)	2,665 (8.6)	379 (9.7)	111 (13.2)	<0.001
Kidney disease	531 (0.2)	89 (0.3)	≤10 (≤0.3)	≤10 (≤1.2)	0.036
Hypertension	19,011 (7.7)	2,372 (7.6)	362 (9.3)	102 (12.2)	<0.001
Diabetes	3,757 (1.5)	486 (1.6)	76 (1.9)	23 (2.7)	0.005
Cardiovascular disease	4,085 (1.7)	1,038 (3.3)	216 (5.5)	74 (8.8)	<0.001

Values are n (%) unless otherwise indicated.

TBI = traumatic brain injury.

Many of the trends were similar in the cohort of men stratified by TBI (Table 3). However, CVD was more common among men at all TBI strata compared to women. Notably, CVD sex differences were largest in the no-TBI group (2.8% of men with no TBI developed CVD compared to 1.7% of women). CVD differences were also present in the mild TBI (3.6% in men compared to 3.3% women), moderate-to-severe TBI (5.9% in men compared to 5.5% women), and penetrating TBI (9.7% in men compared to 8.8% women) groups.

**Table 3:** Characteristics of Males in the Study Cohort

	No TBI (n = 1,012,159, 79.2%)	Mild TBI (n = 220,030, 17.2%)	Moderate or Severe TBI (n = 37,066, 2.9%)	Penetrating TBI (n = 8,175, 0.6%)	*P* Value
Age, y (median, IQR)	29 (24-40)	27 (23-34)	28 (24-35)	29 (24-38)	<0.001
Race and ethnicity					<0.001
Asian/Pacific Islander	58,554 (5.8)	17,871 (8.1)	3,007 (8.1)	666 (8.2)	
Hispanic	96,160 (9.5)	21,357 (9.7)	3,424 (9.2)	793 (9.7)	
Hispanic Black	5,037 (0.5)	970 (0.4)	164 (0.4)	30 (0.4)	
Native American	15,537 (1.5)	4,276 (1.9)	704 (1.9)	146 (1.8)	
Non-Hispanic Black	158,645 (15.7)	28,545 (13.0)	4,791 (12.9)	1,061 (13.0)	
Non-Hispanic White	662,594 (65.5)	145,330 (66.1)	24,795 (66.9)	5,407 (66.1)	
Unknown	15,632 (1.5)	1,681 (0.8)	181 (0.5)	72 (0.9)	
Education					<0.001
Less than high school	14,193 (1.4)	3,445 (1.6)	624 (1.7)	113 (1.4)	
High school	668,870 (66.1)	171,079 (77.8)	28,995 (78.2)	5,937 (72.6)	
>High school	317,540 (31.4)	44,380 (20.2)	7,330 (19.8)	2,083 (25.2)	
Unknown	11,556 (1.1)	1,126 (0.5)	117 (0.3)	42 (0.5)	
Service branch					<0.001
Army	422,387 (41.7)	130,369 (59.3)	22,094 (59.6)	4,844 (59.3)	
Air force	209,106 (20.7)	21,199 (9.6)	3,099 (8.4)	703 (8.6)	
Marines	164,307 (16.2)	42,661 (19.4)	7,649 (20.6)	1,667 (20.4)	
Navy/coast guard	215,548 (21.3)	25,734 (11.7)	4,211 (11.4)	958 (11.7)	
Other	811 (0.1)	67 (0.03)	13 (0.04)	≤10 (≤0.1)	
Component					<0.001
Guard/reserve	497,464 (49.2)	105,777 (48.1)	15,954 (43.0)	2,418 (29.6)	
Active	514,695 (50.9)	114,253 (51.9)	21,112 (57.0)	5,757 (70.4)	
Rank					<0.001
Officer/warrant	154,830 (15.3)	14,838 (6.7)	2,375 (6.4)	749 (9.1)	
Enlisted	857,329 (84.7)	205,192 (93.3)	34,691 (93.6)	7,426 (90.8)	
Deployment history					<0.001
+Combat and deploy	665,860 (65.8)	178,765 (81.3)	30,233 (81.6)	6,399 (78.3)	
+Combat/-deploy	58,242 (5.8)	5,682 (2.6)	1,040 (2.8)	213 (2.6)	
-Combat/+deploy	53,080 (5.2)	8,961 (4.1)	1,346 (3.6)	280 (3.4)	
-Combat or deploy	234,977 (23.2)	26,622 (12.1)	4,447 (12.0)	1,283 (15.7)	
Smoking history	417,016 (41.2)	129,280 (58.8)	22,880 (61.7)	4,867 (59.5)	<0.001
Substance use disorder	108,453 (10.7)	34,351 (15.6)	7,055 (19.0)	1,385 (16.9)	<0.001
Obesity	115,444 (11.4)	25,862 (11.8)	4,940 (13.3)	990 (12.1)	<0.001
Obstructive sleep apnea	62,946 (6.2)	15,094 (6.9)	3,789 (10.2)	845 (10.3)	<0.001
Insomnia	72,447 (7.2)	37,640 (17.1)	8,875 (23.9)	1,874 (22.9)	<0.001
Post-traumatic stress disorder	63,825 (6.3)	59,841 (27.2)	13,573 (36.6)	2,005 (24.5)	<0.001
Depression	103,586 (10.2)	49,832 (22.7)	11,001 (29.7)	1762 (21.6)	<0.001
Anxiety	80,755 (8.0)	40,103 (18.2)	9,311 (25.1)	1,681 (20.6)	<0.001
Hyperlipidemia	169,127 (16.7)	27,683 (12.6)	5,494 (14.8)	1,266 (15.5)	<0.001
Kidney disease	4,751 (0.5)	730 (0.3)	178 (0.5)	37 (0.5)	<0.001
Hypertension	123,683 (12.2)	23,467 (10.7)	4,863 (13.1)	1,054 (12.9)	<0.001
Diabetes	21,724 (2.2)	2,974 (1.4)	675 (1.8)	154 (1.9)	<0.001
Cardiovascular disease	28,522 (2.8)	7,867 (3.6)	2,171 (5.9)	790 (9.7)	<0.001

Values are n (%) unless otherwise indicated.

Abbreviation as in Table 2.

**Figure 2:**
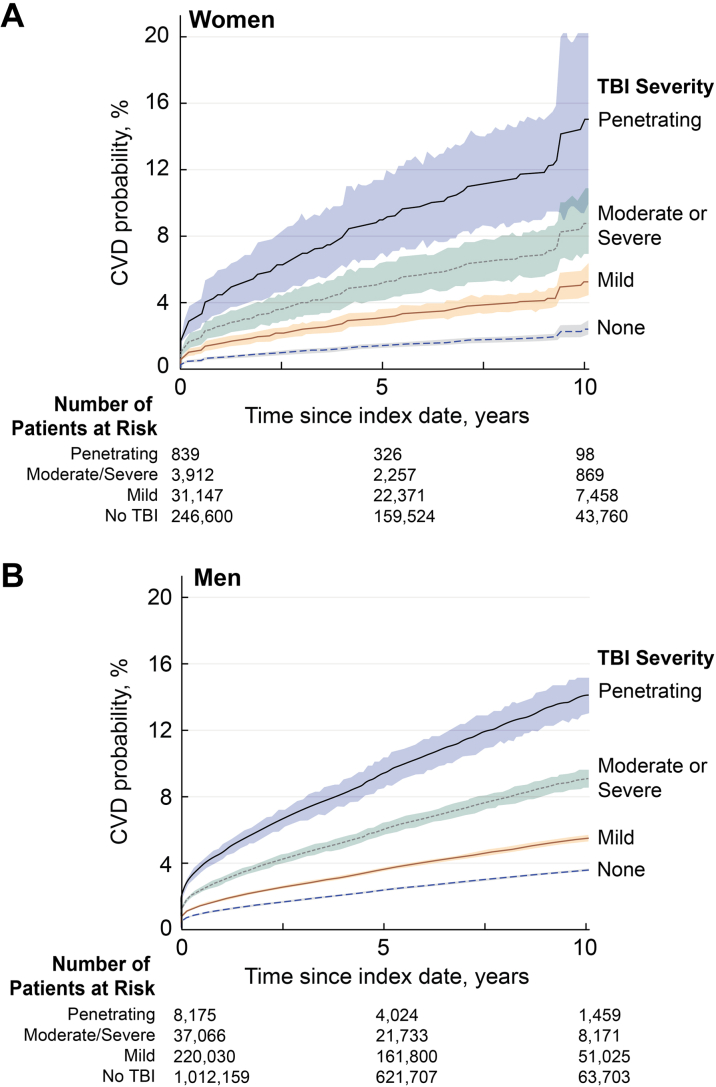
**Cumulative Incidence Functions** Cumulative incidence functions using propensity score weighting for the composite cardiovascular disease outcome stratified by traumatic brain injury severity among women (A) and men (B) after a median follow-up (IQR) of 6.8 years (4.1-9.1) and 6.6 years (3.9-9.0), respectively. Abbreviations as in Figure 1.

Table 4 presents stratified bivariate and multivariable competing risk models of the association between TBI and risk of CVD. These same results are also presented graphically in Supplemental Figure 2. For men, bivariate analysis showed that mild TBI was associated with a 55% increased rate of CVD (HR: 1.55; 95% CI: 1.53-1.57), while moderate/severe and penetrating TBI were associated with a 164% (HR: 2.64; 95% CI: 2.60-2.67) and 321% (HR: 4.21; 95% CI: 4.16-4.26) increased rate, respectively (*P* < 0.001 for all). These trends persisted or increased in fully adjusted models; after adjusting for demographic, military, and health-related covariates, the HRs for mild, moderate/severe, and penetrating TBI were 1.62 (95% CI: 1.60-1.64), 2.65 (95% CI: 2.62-2.69), and 4.22 (95% CI: 4.17-4.27), respectively. Among women, the rate difference was notably higher. In the bivariate model, mild TBI was associated with a 126% increase (HR: 2.26; 95% CI: 2.19-2.34), while moderate/severe and penetrating TBI were associated with a 285% (HR: 3.85; 95% CI: 3.73-3.98) and 586% (HR: 6.86; 95% CI: 6.65-7.07) increased rate, respectively. After adjustment, the HRs remained elevated, with mild TBI at 2.09 (95% CI: 2.02-2.16), moderate/severe TBI at 3.45 (95% CI: 3.34-3.56), and penetrating TBI at 5.61 (95% CI: 5.44-5.78) (*P* < 0.001 for all). Figure 2A shows the CIF for the outcome of CVD in women over time, stratified by TBI severity level. The figure shows a dose-response relationship between TBI severity and risk of developing CVD. Penetrating TBI exhibited the highest cumulative incidence over the 10-year period, followed by moderate-to-severe TBI, mild TBI, and no-TBI groups. Figure 2B illustrates the same CIF plot for men, indicating a similar pattern. CIFs for the composite of CVD outcomes stratified by sex and TBI are presented in Supplemental Figure 1. Nearly all covariates achieved absolute standardized mean differences well below 0.1 and the majority were below 0.05 indicating excellent balance (Supplemental Table 3). Taken together, these results indicate that the weighting successfully balanced observed baseline characteristics across TBI severity groups.

**Table 4:** Bivariate and Multivariable Competing Risk Models for the Outcome of Cardiovascular Disease

	Mild TBI^a^			Moderate/Severe TBI^a^			Penetrating TBI^a^		
	HR	95% CI	*P* Value	HR	95% CI	*P* Value	HR	95% CI	*P* Value
Men									
Bivariate	1.55	1.53-1.57	<0.001	2.64	2.60-2.67	<0.001	4.21	4.16-4.26	<0.001
Adjusted^b^	1.62	1.60-1.64	<0.001	2.65	2.62-2.69	<0.001	4.22	4.17-4.27	<0.001
Women									
Bivariate	2.26	2.19-2.34	<0.001	3.85	3.73-3.98	<0.001	6.86	6.65-7.07	<0.001
Adjusted^b^	2.09	2.02-2.16	<0.001	3.45	3.34-3.56	<0.001	5.61	5.44-5.78	<0.001

Abbreviation as in Table 2.

a Compared to participants without TBI.

b Adjusted for birth year, race and ethnicity, education, service branch, component, rank, deployment history, smoking history, substance use disorder, obesity, depression, anxiety, insomnia, post-traumatic stress disorder, hyperlipidemia, hypertension, kidney disease, diabetes, and obstructive sleep apnea.

Secondary analysis examined the individual components of the composite endpoint (Table 5). In general, women had an increased rate of individual components compared to men. The exceptions to this trend were CAD after moderate/severe TBI (HR: 1.66 [95% CI: 1.64-1.69] and 1.23 [95% CI: 1.17-1.29] in men and women, respectively) and stroke after penetrating TBI (HR: 12.84 [95% CI: 12.53-13.15] in men compared to 10.15 [95% CI: 9.65-10.67] in women). CVD death was only significantly more common in women with moderate/severe TBI; however, it was notably higher than in men with the same TBI severity (HR: 4.21 [95% CI: 3.31-5.36] and 1.67 [95% CI: 1.57-1.77], respectively).

**Table 5:** Multivariable Results for the Individual Components of the Composite Cardiovascular Disease Endpoint: Coronary Artery Disease, Stroke, Peripheral Artery Disease, and Cardiovascular Death

	Mild TBI^a^			Moderate/Severe TBI^a^			Penetrating TBI^a^		
	HR	95% CI	*P* Value	HR	95% CI	*P* Value	HR	95% CI	*P* Value
CAD^b^									
Male	1.41	1.39-1.43	<0.001	1.66	1.64-1.69	<0.001	2.28	2.24-2.31	<0.001
Female	1.93	1.84-2.02	<0.001	1.23	1.17-1.29	<0.001	3.69	3.52-3.86	<0.001
Stroke^b^									
Male	2.52	2.45-2.59	<0.001	6.69	6.53-6.86	<0.001	12.84	12.53-13.15	<0.001
Female	2.70	2.56-2.85	<0.001	6.94	6.60-7.30	<0.001	10.15	9.65-10.67	<0.001
PAD^b^									
Male	1.67	1.64-1.71	<0.001	2.64	2.59-2.70	<0.001	3.62	3.55-3.70	<0.001
Female	1.87	1.77-1.98	<0.001	3.09	2.94-3.26	<0.001	5.17	4.91-5.44	<0.001
CVD death^b^									
Male	1.39	1.31-1.48	<0.001	1.67	1.57-1.77	<0.001	1.01	0.94-1.08	0.790
Female^c^	1.21	0.92-1.60	0.181	4.21	3.31-5.36	<0.001	-	-	-

CAD = coronary artery disease; CVD = cardiovascular disease; PAD = peripheral artery disease; other abbreviation as in Table 2.

a Compared to participants without TBI.

b Adjusted for birth year, race and ethnicity, education, service branch, component, rank, deployment history, smoking history, substance use disorder, obesity, depression, anxiety, insomnia, post-traumatic stress disorder, hyperlipidemia, hypertension, kidney disease, diabetes, and obstructive sleep apnea.

c Model did not converge.

## Discussion

The results of this study demonstrate a significant association between TBI and increased risk of CVD among veterans, with notable sex-based differences in outcomes. While veteran men had a higher prevalence of CVD overall, women with a history of TBI consistently exhibited higher HRs for CVD compared to men across all TBI severity levels (mild, moderate/severe, and penetrating). This result suggests that TBI is a more potent risk factor for CVD in women compared to men. This is despite the fact that traditionally, women have a lower risk of developing CVD. As the number of women service members in combat roles continues to grow, understanding these sex-specific risks will be critical for developing targeted prevention and intervention strategies. Since our study is retrospective in nature, it cannot determine the specific pathophysiology to explain this difference. However, our results are biologically plausible by several mechanisms.

Research suggests that the physiological and psychosocial consequences of TBI may manifest more acutely in women, potentially due to differences in inflammatory responses, hormonal influences, and comorbid mental health conditions following a TBI.^11^ These physiologic and psychosocial consequences could in turn increase CVD risk. For example, there is evidence that women, especially during their childbearing years, may experience a disproportionately higher burden of post-TBI complications, such as post-concussive symptoms and mental health diagnoses.^12^ It has also been shown that women have more severe depression, anxiety, and PTSD symptoms after TBI compared to men.^13^ These high rates of PTSD and other mental health conditions after TBI may increase catecholamines,^14^ hypertension,^15^ endothelial dysfunction,^16^ and inflammation,^17^ leading to subsequent CVD.

Neuroendocrine injury after TBI is another plausible mechanism by which TBI may increase CVD risk in women. Damage to the pituitary gland, leading to hypothalamic-pituitary-gonadal axis disruption, can lower progesterone and estradiol levels,^18^ potentially stripping cardiovascular protections that normally delay CVD in women by ∼7 to 10 years.^19^ Progesterone has protective functions in the cardiovascular system, with evidence that it lowers blood pressure, acts as a vasodilator, and induces endothelium relaxation.^20^ This suggests that a drop in progesterone could play a role in the outcomes of women following TBI. Similarly, estradiol, which is also regulated by the hypothalamic-pituitary-gonadal axis, has been shown to be cardioprotective, improving vascular and mitochondrial function, while also decreasing oxidative stress and fibrosis.^21^ Estradiol’s cardioprotective mechanisms appear to provide protection against CAD and subsequent myocardial infarction by decreasing atherosclerosis and plaque rupture,^22^ but provide less protection against stroke.^23^ With the exception of CAD after moderate/severe TBI, the general trend in our secondary analysis was that TBI was a greater risk factor for CAD than stroke in women compared to men, implying that estradiol deficiency could be playing a role. In our relatively young cohort (largely ages 25-34), loss of these neuroendocrine effects may accelerate traditional risk factors and CVD onset.

Clinically, the finding that a history of TBI, especially in women veterans, confers a significantly higher risk for CVD, suggests incorporating TBI status and severity into routine CVD risk assessment and prevention strategies. Clinicians should consider a history of TBI and TBI severity as factors that may accelerate traditional CVD onset, prompting earlier and more aggressive screening for both traditional risk factors, such as hypertension and hyperlipidemia, as well as TBI-specific risk factors, such as PTSD. In the future, our work suggests the need to develop and validate CVD risk prediction tools that specifically incorporate sex and TBI severity.

## Strengths

The strengths of our study lie in the large cohort of post-9/11-era veterans and the fact that our analysis includes data from both the DoD and VA. This allowed for long-term follow-up of various levels of TBI severity.

### Study Limitations

Limitations of this study include the reliance on retrospective data, which may be subject to misclassification bias, incomplete records, and residual confounding. The date of TBI recorded in the medical records is another limitation, since the date of diagnosis may be different than the actual occurrence of the injury. However, it is important to note that this study employed a thorough definition and operationalization of TBI exposure, incorporating data from multiple sources. Another limitation is that while we had comprehensive data from the DoD and VA health systems, we did not have information on ICD-9-CM and ICD-10-CM codes from private health care insurance providers. This gap is partially offset by the consistency of our findings on CVD mortality, as these data were obtained from NDI and covered all veterans, regardless of their health care provider. The reliance on ICD codes also introduces the risk of misclassification bias. If diagnostic accuracy for CVD following a TBI differs by sex due to variations in health care–seeking behavior or clinical presentation, the reported HRs may overestimate or underestimate the risk in women relative to men. Additionally, 38% of participants from the LIMBIC-CENC Phenotype study were excluded from this analysis. However, most of these exclusions resulted from having an index date after October 1, 2016, which would be unlikely to systematically bias the sample. Another factor to consider is that most of the covariates in this analysis were identified through ICD-9-CM and ICD-10-CM codes. Since ICD codes tend to be more specific than sensitive when detecting cardiovascular risk factors, the baseline prevalence of these conditions may have been underestimated.^24^ Although we adjusted for extensive baseline covariates, we did not adjust for postindex endocrine sequelae (potential mediators of the TBI to CVD pathway) to avoid overadjustment. While not an exclusion criterion, our study did not specifically examine the risk of heart failure. Future work should examine heart failure as an endpoint and evaluate potential pathophysiologic pathways. Lastly, generalizability may be limited. This young, predominantly enlisted, combat-exposed veteran cohort differs from other populations in age, baseline CVD risk, exposures, and access to integrated care, which may impact the generalizability of our findings to civilian and older veteran populations.

## Conclusions

Our results demonstrate that TBI imparts more risk for CVD in women service members compared to men. This effect was consistent across all TBI severity levels and for the most part across the individual components of the composite CVD outcome. More research is needed to examine the potential mechanisms associated with TBI and its effect on traditional CVD risk factors so that we can better understand the relationship between TBI and CVD. These findings also suggest the need for a better understanding of sex-based differences in the long-term health outcomes following TBI and the development of interventions and long-term surveillance to address cardiovascular health in veterans with TBI. These sex-based differences are likely to be important in future conflicts, as the proportion of combat casualties suffered by women is anticipated to increase compared to prior wars given their increased participation in combat roles.

Perspectives **COMPETENCY IN MEDICAL KNOWLEDGE:** In post-9/11 veterans, TBI is associated with increased composite CVD risk; women exhibit higher relative hazards than men at every TBI severity level.

**COMPETENCY IN PATIENT CARE:** Incorporate TBI history—especially in women—into routine CVD risk assessment and prevention with attention to TBI severity.

**TRANSLATIONAL OUTLOOK 1:** Define potential sex-specific mechanisms (eg, neuroendocrine, autonomic, mental health pathways) linking TBI to CVD that might enable future targeted therapies.

**TRANSLATIONAL OUTLOOK 2:** Develop and validate TBI-informed CVD risk prediction tools that incorporate sex and TBI severity for use in VA/DoD electronic health records.

### Disclaimer

The opinions and assertions expressed herein are those of the author(s) and do not necessarily reflect the official policy or position of the Uniformed Services University, the Department of Defense, or the Department of Veterans Affairs.

## Funding support and author disclosures

This work was supported by the Assistant Secretary of Defense for Health Affairs, endorsed by the Department of Defense through the Psychological Health/Traumatic Brain Injury Research Program Long-Term Impact of Military-Relevant Brain Injury Consortium (LIMBIC) Award/W81XWH-18-PH/TBIRP-LIMBIC under Award Nos. W81XWH1920067 and W81XWH-13-2-0095, and by the U.S. Department of Veterans Affairs Award No. I01 RX003443. The U.S. Army Medical Research Acquisition Activity, 839 Chandler Street, Fort Detrick MD 21702-5014 is the awarding and administering acquisition office. Dr Pugh’s effort was also funded by VA Health Services Research and Development Service Award No. IHX002608A and supported in part by the VA HSR&D Informatics, Decision-Enhancement, and Analytic Sciences (IDEAS) Center of Innovation (CIN 13-414). The Defense Health Agency also provided funding, under award HU0001232000. The funders had no role in the design and conduct of the study; collection, management, analysis, and interpretation of the data; preparation, review, or approval of the manuscript; and decision to submit the manuscript for publication. The authors have reported that they have no relationships relevant to the contents of this paper to disclose.
