# Supplementary material for: Sex Differences in Cardiovascular Disease Outcomes After Traumatic Brain Injury
Source: JACC Adv. 2026 Mar 18;5(4):102658. doi: 10.1016/j.jacadv.2026.102658 (PMC13010400; doi:10.1016/j.jacadv.2026.102658)

Supplemental Table 1. International Classification of Diseases 9^th^ and 10^th^ Edition Clinical Modification Codes Used to Determine Variables

|  | ICD9 | ICD10 |
| --- | --- | --- |
| Diabetes | UNCOMPLICATED  250.0X, 250.1X, 250.2X,250.3X  COMPLICATED  250.4X, 250.5X, 250.6X, 250.7X, 250.8X, 250.9X | UNCOMPLICATED  E100, E101, E109, E110, E111, E119, E120, E121, E129, E130, E131, E139, E140, E141, E149  COMPLICATED  E102, E103, E104, E105, E106, E107, E108, E112, E113, E114, E115, E116, E117, E118, E122, E123, E124, E125, E126, E127, E128, E132, E133, E134, E135, E136, E137, E138, E142, E143, E144, E145, E146,  E147, E148 |
| Hypertension | UNCOMPLICATED  401.XX  COMPLICATED  402.XX, 403.XX, 404.XX, 405.XX | UNCOMPLICATED  I10  COMPLICATED  I11, I12, I13, I15 |
| Hyperlipidemia | 272.0X, 272.1X, 272.2X, 272.3X, 272.4X | E780, E781, E782, E783, E784, E785 |
| Depression | 296.2X, 296.3X, 311.XX | F32, F33 AND NOT F328 |
| Anxiety | 300.0X, 300.2X, 300.3X | F40, F41, F42 |
| Kidney Disease | 403.01, 403.11, 403.91, 404.02, 404.03, 404.12, 404.13, 404.92, 404.93, 585.XX, 586.XX, 588.0X, V42.0X, V45.1X, V56.XX | I120, I131, N18, N19, N250, Z490, Z491, Z492, Z940, Z992 |
| Insomnia | 780.51, 780.52, 327.0X, 307.4X | F5101, F5102, F5103, F5104, F5105, F5109, G4700, G4701, G4709 |
| Obstructive Sleep Apnea | 327.23 | G473 |
| Obesity | 278.0X | E40, E41, E42, E43, E44, E45, E46, R634, R64 |
| Substance Use Disorder | Opioid  304.0X, 304.7X, 305.5X, 965.0X, E8500, E9350  Alcohol  291.0X, 291.1X, 291.2X, 291.3X, 291.4X, 291.5X, 291.8X, 291.9X, 303.0X, 303.9X, 305.0X, 357.5X, 425.5X, 535.3X, 571.0X, 571.1X,571.2X, 571.3X, E8600, 980XX, V113X  Amphetamine  304.4X, 305.7X  Cannabis  304.3X, 305.2X  Sedative  304.1X, 305.4X  Cocaine  304.2X, 305.6X, 968.5X, E9385  Hallucinogens  304.5X, 305.3X, 969.6X, E8541, E9396  Other Abuse  304.6X, 304.8X, 304.9X, 305.9X, 648.3X, V6542  Drug Induced Mental  292.0x, 292.11, 292.12, 292.2X, 292.81, 292.82, 292.83, 292.84, 292.85, 292.89, 292.9X | Opioid  F11, T400, T401, T402, T403  Alcohol  F10, G621, I426, K2920, K2921, K700, K7010, K709, T51, Z7141  Amphetamine  F15  Cannabis  F12, T407  Sedative  F13  Cocaine  F14, T405  Hallucinogens  F16, T408, T409  Other Abuse  F18, F19, O9932x  Drug Induced Mental  F11159, F11181, F11182, F11188, F11222, F11259, F11281,  F11282, F11288, F11922, F11959, F11981, F11982, F11988,  F12122, F12159,F12180, F12188, F12222, F12259, F12280,  F12288, F12922, F12959, F12980, F12988, F13159, F13180,  F13181, F13182, F13188, F13259,F13280, F13281, F13282,  F13288, F13959, F13980, F13981, F13982, F13988, F14122,  F14159, F14180, F14181, F14182, F14188, F14222, F14259,  F14280, F14281, F14282, F14288, F14922, F14959, F14980,  F14981, F14982, F14988, F15122, F15159, F15180, F15181,  F15182, F15188, F15222, F15259, F15280, F15281, F15282,  F15288, F15920, F15922, F15959, F15980, F15981, F15982,  F15988, F16122, F16159, F16180, F16183, F16188, F16259,  F16280, F16283, F16288, F16959, F16980, F16983, F16988,  F17208, F17218, F17228, F17298, F18159, F18180, F18188,  F18259, F18280, F18288, F18959, F18980, F18988, F19122,  F19159, F19180, F19181, F19182, F19188, F19222, F19259,  F19280, F19281, F19282, F19288, F19921, F19922, F19939,  F1994, F19950, F19951, F19959, F1996, F1997, F19980, F19981,  F19982, F19988, F1999 |
| Post-traumatic Stress Disorder | 309.81 | F4310, F4312 |
| Cardiovascular Disease | Acute coronary heart disease  410.X-414.X (excluding 412)  Previous coronary heart disease  412  Cardiac arrest  427.5  Ischemic stroke, transient ischemic attack, or documented atherosclerotic  cerebrovascular disease  434.X, 436.X, 437.1, 437.3, 438.X, 435.X  Peripheral arterial disease (PAD)  433.X, 441.X, 442.X, 444.X, 443.9, 440.21- 440.24  Coronary procedures  360.X, 361.X, 362.X  Peripheral arterial procedures  380.X, 381.X, 392.2-392.6,  392.8 | Acute coronary heart disease  I20X to I25X (excluding I252)  Previous coronary heart disease  I252  Cardiac arrest  I46X, R96, R98  Ischemic stroke, transient ischemic attack, or documented atherosclerotic  cerebrovascular disease  I63X, I64X, I66X, I670, I671,  I693, I694, I698, G45X  (except G453), G46X  Peripheral arterial disease (PAD)  I65X, I71X, I72X, I74X, I739,  I7021, E1051, E1052, E1151,  E1152, E1451, E1452  Coronary procedures  0210098, 0210099, 021009C, 021009F, 021009W, 02100A3, 02100A8, 02100A9, 02100AC, 02100AF, 02100AW, 02100J3, 02100J8, 02100J9, 02100JC, 02100JF, 02100JW, 02100K3, 02100K8, 02100K9, 02100KC, 02100KF, 02100KW, 02100Z3, 02100Z8, 02100Z9, 02100ZC, 02100ZF, 0210493, 0210498, 0210499, 021049C, 021049F, 021049W, 02104A3, 02104A8, 02104A9, 02104AC, 02104AF,  02104AW, 02104J3, 02104J8,  02104J9, 02104JC, 02104JF,  02104JW, 02104K3, 02104K8, 02104K9, 02104KC, 02104KF,  02104KW, 02104Z3, 02104Z8,  02104Z9, 02104ZC, 02104ZF,  0211098, 0211099, 021109C,  021109W, 02110A8, 02110A9,  02110AC, 02110AW, 02110J8,  02110J9, 02110JC, 02110JW,  02110K8, 02110K9, 02110KC,  02110KW, 02110Z8, 02110Z9,  02110ZC, 0211498, 0211499,  021149C, 021149W, 02114A8, 02114A9, 02114AC, 02114AW,  02114J8, 02114J9, 02114JC,  02114JW, 02114K8, 02114K9,  02114KC, 02114KW, 02114Z8,  02114Z9, 02114ZC, 021209C,  021209W, 02120AC, 02120AW,  02120JC, 02120JW, 02120KC,  02120KW, 02120ZC, 021249C,  021249W, 02124AC, 02124AW,  02124JC, 02124JW, 02124KC,  02124KW, 02124ZC, 021309C,  021309W, 02130AC, 02130AW,  02130JC, 02130JW, 02130KC,  02130KW, 02130ZC, 021349C,  021349W, 02134AC, 02134AW,  02134JC, 02134JW, 02134KC,  02134KW, 02134ZC, 021K0Z8,  021K0Z9, 021K0ZC, 021K0ZW,  021K4Z8, 021K4Z9, 021K4ZC,  021K4ZW, 021L0Z8, 021L0Z9,  021L0ZC, 021L4Z8, 021L4Z9,  021L4ZC, 02700ZZ, 02710ZZ,  02720ZZ, 02730ZZ, 02C00ZZ,  02C03ZZ, 02C04ZZ, 02C10ZZ,  02C13ZZ, 02C14ZZ, 02C20ZZ,  02C23ZZ, 02C24ZZ, 02C30ZZ,  02C33ZZ, 02C34ZZ, 3E07017,  3E070PZ, 3E07317, 3E073PZ, 021W09B, 021W09D, 021W0AB,  021W0AD, 021W0JB, 021W0JD,  021W0KB, 021W0KD, 021W0ZB, 021W0ZD, 021W49B,  021W49D, 021W4AB, 021W4AD, 021W4JB, 021W4JD,  021W4KB, 021W4KD, 021W4ZB, 021W4ZD, 02CP0ZZ,  02CP3ZZ, 02CP4ZZ, 02CQ0ZZ,  02CQ3ZZ, 02CQ4ZZ, 02CR0ZZ,  02CR3ZZ, 02CR4ZZ, 02CS0ZZ,  02CS3ZZ, 02CS4ZZ, 02CT0ZZ,  02CT3ZZ, 02CT4ZZ, 02CV0ZZ,  02CV3ZZ, 02CV4ZZ, 02CW0ZZ,  02CW3ZZ, 02CW4ZZ, 02HP0DZ,  02HP3DZ, 02HP4DZ, 02HQ0DZ,  02HQ3DZ, 02HQ4DZ, 02HR0DZ,  02HR3DZ, 02HR4DZ, 02HS02Z,  02HS0DZ, 02HS32Z, 02HS3DZ,  02HS42Z, 02HS4DZ, 02HT02Z,  02HT0DZ, 02HT32Z, 02HT3DZ,  02HT42Z, 02HT4DZ, 02HW02Z,  02HW0DZ, 02HW32Z, 02HW3DZ, 02HW42Z, 02HW4DZ, 031H09J, 031H0AJ,  031H0JJ, 031H0KJ, 031H0ZJ,  031J09K, 031J0AK, 031J0JK,  031J0KK, 031J0ZK, 031K09J,  031K0AJ, 031K0JJ, 031K0KJ, 031K0ZJ, 031L09K, 031L0AK,  031L0JK, 031L0KK, 031L0ZK,  031M09J, 031M0AJ, 031M0JJ,  031M0KJ, 031M0ZJ, 031N09K,  031N0AK, 031N0JK, 031N0KK,  031N0ZK, 031S09G, 031S0AG,  031S0JG, 031S0KG, 031S0ZG,  031T09G, 031T0AG, 031T0JG,  031T0KG, 031T0ZG, 039Y0ZZ,  039Y3ZZ, 039Y4ZZ, 03C00ZZ,  03C03ZZ, 03C04ZZ, 03C10ZZ,  03C13ZZ, 03C14ZZ, 03C20ZZ,  03C23ZZ, 03C24ZZ, 03C30ZZ,  03C33ZZ, 03C34ZZ, 03C40ZZ,  03C43ZZ, 03C44ZZ, 03C50ZZ,  03C53ZZ, 03C54ZZ, 03C60ZZ,  03C63ZZ, 03C64ZZ, 03C70ZZ,  03C73ZZ, 03C74ZZ, 03C80ZZ,  03C83ZZ, 03C84ZZ, 03C90ZZ,  03C93ZZ, 03C94ZZ, 03CA0ZZ,  03CA3ZZ, 03CA4ZZ, 03CB0ZZ,  03CB3ZZ, 03CB4ZZ, 03CC0ZZ,  03CC3ZZ, 03CC4ZZ, 03CD0ZZ,  03CD3ZZ, 03CD4ZZ, 03CF0ZZ,  03CF3ZZ, 03CF4ZZ, 03CG0ZZ,  03CG4ZZ, 03CH0ZZ, 03CH4ZZ,  03CJ0ZZ, 03CJ4ZZ, 03CK0ZZ,  03CK4ZZ, 03CL0ZZ, 03CL4ZZ,  03CM0ZZ, 03CM4ZZ, 03CN0ZZ,  03CN4ZZ, 03CP0ZZ, 03CP4ZZ,  03CQ0ZZ, 03CQ4ZZ, 03CR0ZZ,  03CR3ZZ, 03CR4ZZ, 03CS0ZZ,  03CS3ZZ, 03CS4ZZ, 03CT0ZZ,  03CT3ZZ, 03CT4ZZ, 03CU0ZZ,  03CU3ZZ, 03CU4ZZ, 03CV0ZZ,  03CV3ZZ, 03CV4ZZ, 03CY0ZZ,  03CY3ZZ, 03CY4ZZ, 03HY02Z,  03HY32Z, 03HY42Z, 0410090,  0410091, 0410092, 0410093,  0410094, 0410095, 0410096,  0410097, 0410098, 0410099, 041009B, 041009C, 041009D,  041009F, 041009G, 041009H,  041009J, 041009K, 041009Q,  041009R, 04100A0, 04100A1,  04100A2, 04100A3, 04100A4,  04100A5, 04100A6, 04100A7,  04100A8, 04100A9, 04100AB,  04100AC, 04100AD, 04100AF,  04100AG, 04100AH, 04100AJ,  04100AK, 04100AQ, 04100AR,  04100J0, 04100J1, 04100J2,  04100J3, 04100J4, 04100J5,  04100J6, 04100J7, 04100J8,  04100J9, 04100JB, 04100JC,  04100JD, 04100JF, 04100JG,  04100JH, 04100JJ, 04100JK,  04100JQ, 04100JR, 04100K0,  04100K1, 04100K2, 04100K3,  04100K4, 04100K5, 04100K6,  04100K7, 04100K8, 04100K9,  04100KB, 04100KC, 04100KD,  04100KF, 04100KG, 04100KH,  04100KJ, 04100KK, 04100KQ,  04100KR, 04100Z0, 04100Z1,  04100Z2, 04100Z3, 04100Z4,  04100Z5, 04100Z6, 04100Z7,  04100Z8, 04100Z9, 04100ZB,  04100ZC, 04100ZD, 04100ZF,  04100ZG, 04100ZH, 04100ZJ,  04100ZK, 04100ZQ, 04100ZR,  0410490, 0410491, 0410492,  0410493, 0410494, 0410495,  0410496, 0410497, 0410498,  0410499, 041049B, 041049C,  041049D, 041049F, 041049G,  041049H, 041049J, 041049K,  041049Q, 041049R, 04104A0,  04104A1, 04104A2, 04104A3,  04104A4, 04104A5, 04104A6,  04104A7, 04104A8, 04104A9,  04104AB, 04104AC, 04104AD, 04104AF, 04104AG, 04104AH,  04104AJ, 04104AK, 04104AQ,  04104AR, 04104J0, 04104J1,  04104J2, 04104J3, 04104J4,  04104J5, 04104J6, 04104J7,  04104J8, 04104J9, 04104JB,  04104JC, 04104JD, 04104JF,  04104JG, 04104JH, 04104JJ,  04104JK, 04104JQ, 04104JR,  04104K0, 04104K1, 04104K2,  04104K3, 04104K4, 04104K5,  04104K6, 04104K7, 04104K8,  04104K9, 04104KB, 04104KC,  04104KD, 04104KF, 04104KG,  04104KH, 04104KJ, 04104KK,  04104KQ, 04104KR, 04104Z0,  04104Z1, 04104Z2, 04104Z3,  04104Z4, 04104Z5, 04104Z6,  04104Z7, 04104Z8, 04104Z9,  04104ZB, 04104ZC, 04104ZD,  04104ZF, 04104ZG, 04104ZH,  04104ZJ, 04104ZK, 04104ZQ,  04104ZR, 0414093, 0414094,  0414095, 04140A3, 04140A4,  04140A5, 04140J3, 04140J4,  04140J5, 04140K3, 04140K4,  04140K5, 04140Z3, 04140Z4,  04140Z5, 0414493, 0414494,  0414495, 04144A3, 04144A4,  04144A5, 04144J3, 04144J4,  04144J5, 04144K3, 04144K4,  04144K5, 04144Z3, 04144Z4,  04144Z5, 041C090, 041C091,  041C092, 041C093, 041C094,  041C095, 041C096, 041C097,  041C098, 041C099, 041C09B,  041C09C, 041C09D, 041C09F,  041C09G, 041C09H, 041C09J,  041C09K, 041C09Q, 041C09R,  041C0A0, 041C0A1, 041C0A2,  041C0A3, 041C0A4, 041C0A5, 041C0A6, 041C0A7, 041C0A8,  041C0A9, 041C0AB, 041C0AC,  041C0AD, 041C0AF, 041C0AG,  041C0AH, 041C0AJ, 041C0AK,  041C0AQ, 041C0AR, 041C0J0,  041C0J1, 041C0J2, 041C0J3,  041C0J4, 041C0J5, 041C0J6,  041C0J7, 041C0J8, 041C0J9,  041C0JB, 041C0JC, 041C0JD,  041C0JF, 041C0JG, 041C0JH,  041C0JJ, 041C0JK, 041C0JQ,  041C0JR, 041C0K0, 041C0K1,  041C0K2, 041C0K3, 041C0K4,  041C0K5, 041C0K6, 041C0K7,  041C0K8, 041C0K9, 041C0KB,  041C0KC, 041C0KD, 041C0KF,  041C0KG, 041C0KH, 041C0KJ,  041C0KK, 041C0KQ, 041C0KR,  041C0Z0, 041C0Z1, 041C0Z2,  041C0Z3, 041C0Z4, 041C0Z5,  041C0Z6, 041C0Z7, 041C0Z8,  041C0Z9, 041C0ZB, 041C0ZC,  041C0ZD, 041C0ZF, 041C0ZG,  041C0ZH, 041C0ZJ, 041C0ZK,  041C0ZQ, 041C0ZR, 041C490,  041C491, 041C492, 041C493,  041C494, 041C495, 041C496,  041C497, 041C498, 041C499,  041C49B, 041C49C, 041C49D,  041C49F, 041C49G, 041C49H,  041C49J, 041C49K, 041C49Q,  041C49R, 041C4A0, 041C4A1,  041C4A2, 041C4A3, 041C4A4,  041C4A5, 041C4A6, 041C4A7,  041C4A8, 041C4A9, 041C4AB,  041C4AC, 041C4AD, 041C4AF,  041C4AG, 041C4AH, 041C4AJ,  041C4AK, 041C4AQ, 041C4AR,  041C4J0, 041C4J1, 041C4J2,  041C4J3, 041C4J4, 041C4J5,  041C4J6, 041C4J7, 041C4J8, 041C4J9, 041C4JB, 041C4JC,  041C4JD, 041C4JF, 041C4JG,  041C4JH, 041C4JJ, 041C4JK,  041C4JQ, 041C4JR, 041C4K0,  041C4K1, 041C4K2, 041C4K3,  041C4K4, 041C4K5, 041C4K6,  041C4K7, 041C4K8, 041C4K9,  041C4KB, 041C4KC, 041C4KD,  041C4KF, 041C4KG, 041C4KH,  041C4KJ, 041C4KK, 041C4KQ,  041C4KR, 041C4Z0, 041C4Z1,  041C4Z2, 041C4Z3, 041C4Z4,  041C4Z5, 041C4Z6, 041C4Z7,  041C4Z8, 041C4Z9, 041C4ZB,  041C4ZC, 041C4ZD, 041C4ZF,  041C4ZG, 041C4ZH, 041C4ZJ,  041C4ZK, 041C4ZQ, 041C4ZR,  041D090, 041D091, 041D092,  041D093, 041D094, 041D095,  041D096, 041D097, 041D098,  041D099, 041D09B, 041D09C,  041D09D, 041D09F, 041D09G,  041D09H, 041D09J, 041D09K,  041D09Q, 041D09R, 041D0A0,  041D0A1, 041D0A2, 041D0A3,  041D0A4, 041D0A5, 041D0A6,  041D0A7, 041D0A8, 041D0A9,  041D0AB, 041D0AC, 041D0AD,  041D0AF, 041D0AG, 041D0AH,  041D0AJ, 041D0AK, 041D0AQ,  041D0AR, 041D0J0, 041D0J1,  041D0J2, 041D0J3, 041D0J4,  041D0J5, 041D0J6, 041D0J7,  041D0J8, 041D0J9, 041D0JB,  041D0JC, 041D0JD, 041D0JF,  041D0JG, 041D0JH, 041D0JJ,  041D0JK, 041D0JQ, 041D0JR,  041D0K0, 041D0K1, 041D0K2,  041D0K3, 041D0K4, 041D0K5,  041D0K6, 041D0K7, 041D0K8,  041D0K9, 041D0KB, 041D0KC, 041D0KD, 041D0KF, 041D0KG,  041D0KH, 041D0KJ, 041D0KK,  041D0KQ, 041D0KR, 041D0Z0,  041D0Z1, 041D0Z2, 041D0Z3,  041D0Z4, 041D0Z5, 041D0Z6,  041D0Z7, 041D0Z8, 041D0Z9,  041D0ZB, 041D0ZC, 041D0ZD,  041D0ZF, 041D0ZG, 041D0ZH,  041D0ZJ, 041D0ZK, 041D0ZQ,  041D0ZR, 041D490, 041D491,  041D492, 041D493, 041D494,  041D495, 041D496, 041D497,  041D498, 041D499, 041D49B,  041D49C, 041D49D, 041D49F,  041D49G, 041D49H, 041D49J,  041D49K, 041D49Q, 041D49R,  041D4A0, 041D4A1, 041D4A2,  041D4A3, 041D4A4, 041D4A5,  041D4A6, 041D4A7, 041D4A8,  041D4A9, 041D4AB, 041D4AC,  041D4AD, 041D4AF, 041D4AG,  041D4AH, 041D4AJ, 041D4AK,  041D4AQ, 041D4AR, 041D4J0,  041D4J1, 041D4J2, 041D4J3,  041D4J4, 041D4J5, 041D4J6,  041D4J7, 041D4J8, 041D4J9,  041D4JB, 041D4JC, 041D4JD,  041D4JF, 041D4JG, 041D4JH,  041D4JJ, 041D4JK, 041D4JQ,  041D4JR, 041D4K0, 041D4K1,  041D4K2, 041D4K3, 041D4K4,  041D4K5, 041D4K6, 041D4K7,  041D4K8, 041D4K9, 041D4KB,  041D4KC, 041D4KD, 041D4KF,  041D4KG, 041D4KH, 041D4KJ,  041D4KK, 041D4KQ, 041D4KR,  041D4Z0, 041D4Z1, 041D4Z2,  041D4Z3, 041D4Z4, 041D4Z5,  041D4Z6, 041D4Z7, 041D4Z8,  041D4Z9, 041D4ZB, 041D4ZC,  041D4ZD, 041D4ZF, 041D4ZG, 041D4ZH, 041D4ZJ, 041D4ZK,  041D4ZQ, 041D4ZR,041E099,  041E09B, 041E09C, 041E09D,  041E09F, 041E09G, 041E09H,  041E09J, 041E09K, 041E09P,  041E09Q, 041E0A9, 041E0AB,  041E0AC, 041E0AD, 041E0AF,  041E0AG, 041E0AH, 041E0AJ,  041E0AK, 041E0AP, 041E0AQ,  041E0J9, 041E0JB, 041E0JC,  041E0JD, 041E0JF, 041E0JG,  041E0JH, 041E0JJ, 041E0JK,  041E0JP, 041E0JQ, 041E0K9,  041E0KB, 041E0KC, 041E0KD,  041E0KF, 041E0KG, 041E0KH,  041E0KJ, 041E0KK, 041E0KP,  041E0KQ, 041E0Z9, 041E0ZB,  041E0ZC, 041E0ZD, 041E0ZF,  041E0ZG, 041E0ZH, 041E0ZJ,  041E0ZK, 041E0ZP, 041E0ZQ,  041E499, 041E49B, 041E49C,  041E49D, 041E49F, 041E49G,  041E49H, 041E49J, 041E49K,  041E49P, 041E49Q, 041E4A9,  041E4AB, 041E4AC, 041E4AD,  041E4AF, 041E4AG, 041E4AH,  041E4AJ, 041E4AK, 041E4AP,  041E4AQ, 041E4J9, 041E4JB,  041E4JC, 041E4JD, 041E4JF,  041E4JG, 041E4JH, 041E4JJ,  041E4JK, 041E4JP, 041E4JQ,  041E4K9, 041E4KB, 041E4KC,  041E4KD, 041E4KF, 041E4KG,  041E4KH, 041E4KJ, 041E4KK,  041E4KP, 041E4KQ, 041E4Z9,  041E4ZB, 041E4ZC, 041E4ZD,  041E4ZF, 041E4ZG, 041E4ZH,  041E4ZJ, 041E4ZK, 041E4ZP,  041E4ZQ, 041F099, 041F09B,  041F09C, 041F09D, 041F09F,  041F09G, 041F09H, 041F09J, 041F09K, 041F09P, 041F09Q,  041F0A9, 041F0AB, 041F0AC,  041F0AD, 041F0AF, 041F0AG,  041F0AH, 041F0AJ, 041F0AK,  041F0AP, 041F0AQ, 041F0J9,  041F0JB, 041F0JC, 041F0JD,  041F0JF, 041F0JG, 041F0JH,  041F0JJ, 041F0JK, 041F0JP,  041F0JQ, 041F0K9, 041F0KB,  041F0KC, 041F0KD, 041F0KF,  041F0KG, 041F0KH, 041F0KJ,  041F0KK, 041F0KP, 041F0KQ,  041F0Z9, 041F0ZB, 041F0ZC,  041F0ZD, 041F0ZF, 041F0ZG,  041F0ZH, 041F0ZJ, 041F0ZK,  041F0ZP, 041F0ZQ, 041F499,  041F49B, 041F49C, 041F49D,  041F49F, 041F49G, 041F49H,  041F49J, 041F49K, 041F49P,  041F49Q, 041F4A9, 041F4AB,  041F4AC, 041F4AD, 041F4AF,  041F4AG, 041F4AH, 041F4AJ,  041F4AK, 041F4AP, 041F4AQ,  041F4J9, 041F4JB, 041F4JC,  041F4JD, 041F4JF, 041F4JG,  041F4JH, 041F4JJ, 041F4JK,  041F4JP, 041F4JQ, 041F4K9,  041F4KB, 041F4KC, 041F4KD,  041F4KF, 041F4KG, 041F4KH,  041F4KJ, 041F4KK, 041F4KP,  041F4KQ, 041F4Z9, 041F4ZB,  041F4ZC, 041F4ZD, 041F4ZF,  041F4ZG, 041F4ZH, 041F4ZJ,  041F4ZK, 041F4ZP, 041F4ZQ,  041H099, 041H09B, 041H09C,  041H09D, 041H09F, 041H09G,  041H09H, 041H09J, 041H09K,  041H09P, 041H09Q, 041H0A9,  041H0AB, 041H0AC, 041H0AD,  041H0AF, 041H0AG, 041H0AH,  041H0AJ, 041H0AK, 041H0AP, 041H0AQ, 041H0J9, 041H0JB,  041H0JC, 041H0JD, 041H0JF,  041H0JG, 041H0JH, 041H0JJ,  041H0JK, 041H0JP, 041H0JQ,  041H0K9, 041H0KB, 041H0KC,  041H0KD, 041H0KF, 041H0KG,  041H0KH, 041H0KJ, 041H0KK,  041H0KP, 041H0KQ, 041H0Z9,  041H0ZB, 041H0ZC, 041H0ZD,  041H0ZF, 041H0ZG, 041H0ZH,  041H0ZJ, 041H0ZK, 041H0ZP,  041H0ZQ, 041H499, 041H49B,  041H49C, 041H49D, 041H49F,  041H49G, 041H49H, 041H49J,  041H49K, 041H49P, 041H49Q,  041H4A9, 041H4AB, 041H4AC,  041H4AD, 041H4AF, 041H4AG,  041H4AH, 041H4AJ, 041H4AK,  041H4AP, 041H4AQ, 041H4J9,  041H4JB, 041H4JC, 041H4JD,  041H4JF, 041H4JG, 041H4JH,  041H4JJ, 041H4JK, 041H4JP,  041H4JQ, 041H4K9, 041H4KB,  041H4KC, 041H4KD, 041H4KF,  041H4KG, 041H4KH, 041H4KJ,  041H4KK, 041H4KP, 041H4KQ,  041H4Z9, 041H4ZB, 041H4ZC,  041H4ZD, 041H4ZF, 041H4ZG,  041H4ZH, 041H4ZJ, 041H4ZK,  041H4ZP, 041H4ZQ, 041J099,  041J09B, 041J09C, 041J09D,  041J09F, 041J09G, 041J09H,  041J09J, 041J09K, 041J09P,  041J09Q, 041J0A9, 041J0AB,  041J0AC, 041J0AD, 041J0AF,  041J0AG, 041J0AH, 041J0AJ,  041J0AK, 041J0AP, 041J0AQ,  041J0J9, 041J0JB, 041J0JC,  041J0JD, 041J0JF, 041J0JG,  041J0JH, 041J0JJ, 041J0JK,  041J0JP, 041J0JQ, 041J0K9, 041J0KB, 041J0KC, 041J0KD,  041J0KF, 041J0KG, 041J0KH,  041J0KJ, 041J0KK, 041J0KP,  041J0KQ, 041J0Z9, 041J0ZB,  041J0ZC, 041J0ZD, 041J0ZF,  041J0ZG, 041J0ZH, 041J0ZJ,  041J0ZK, 041J0ZP, 041J0ZQ,  041J499, 041J49B, 041J49C,  041J49D, 041J49F, 041J49G,  041J49H, 041J49J, 041J49K,  041J49P, 041J49Q, 041J4A9,  041J4AB, 041J4AC, 041J4AD,  041J4AF, 041J4AG, 041J4AH,  041J4AJ, 041J4AK, 041J4AP,  041J4AQ, 041J4J9, 041J4JB,  041J4JC, 041J4JD, 041J4JF,  041J4JG, 041J4JH, 041J4JJ,  041J4JK, 041J4JP, 041J4JQ,  041J4K9, 041J4KB, 041J4KC,  041J4KD, 041J4KF, 041J4KG,  041J4KH, 041J4KJ, 041J4KK,  041J4KP, 041J4KQ, 041J4Z9,  041J4ZB, 041J4ZC, 041J4ZD,  041J4ZF, 041J4ZG, 041J4ZH,  041J4ZJ, 041J4ZK, 041J4ZP,  041J4ZQ, 049Y0ZZ, 049Y3ZZ,  049Y4ZZ, 04C00ZZ, 04C03ZZ,  04C04ZZ,04C10ZZ, 04C13ZZ,  04C14ZZ, 04C20ZZ, 04C23ZZ,  04C24ZZ, 04C30ZZ, 04C33ZZ,  04C34ZZ, 04C40ZZ, 04C43ZZ,  04C44ZZ, 04C50ZZ, 04C53ZZ,  04C54ZZ, 04C60ZZ, 04C63ZZ,  04C64ZZ, 04C70ZZ, 04C73ZZ,  04C74ZZ, 04C80ZZ, 04C83ZZ,  04C84ZZ, 04C90ZZ, 04C93ZZ,  04C94ZZ, 04CA0ZZ, 04CA3ZZ,  04CA4ZZ, 04CB0ZZ, 04CB3ZZ,  04CB4ZZ, 04CC0ZZ, 04CC3ZZ,  04CC4ZZ, 04CD0ZZ, 04CD3ZZ,  04CD4ZZ, 04CE0ZZ, 04CE3ZZ, 04CE4ZZ, 04CF0ZZ, 04CF3ZZ,  04CF4ZZ, 04CH0ZZ, 04CH3ZZ, 04CH4ZZ, 04CJ0ZZ, 04CJ3ZZ,  04CJ4ZZ, 04CK0ZZ, 04CK3ZZ,  04CK4ZZ, 04CL0ZZ, 04CL3ZZ,  04CL4ZZ, 04CM0ZZ, 04CM3ZZ,  04CM4ZZ, 04CN0ZZ, 04CN3ZZ,  04CN4ZZ, 04CP0ZZ, 04CP3ZZ,  04CP4ZZ, 04CQ0ZZ, 04CQ3ZZ,  04CQ4ZZ, 04CR0ZZ, 04CR3ZZ,  04CR4ZZ, 04CS0ZZ, 04CS3ZZ,  04CS4ZZ, 04CT0ZZ, 04CT3ZZ,  04CT4ZZ, 04CU0ZZ, 04CU3ZZ,  04CU4ZZ, 04CV0ZZ, 04CV3ZZ,  04CV4ZZ, 04CW0ZZ, 04CW3ZZ,  04CW4ZZ, 04CY0ZZ, 04CY3ZZ,  04CY4ZZ, 04HY02Z, 04HY32Z,  04HY42Z, 051007Y, 051009Y,  05100AY, 05100JY, 05100KY,  05100ZY, 051047Y, 051049Y,  05104AY, 05104JY, 05104KY,  05104ZY, 051107Y, 051109Y,  05110AY, 05110JY, 05110KY,  05110ZY, 051147Y, 051149Y,  05114AY, 05114JY, 05114KY,  05114ZY, 051307Y, 051309Y,  05130AY, 05130JY, 05130KY,  05130ZY, 051347Y, 051349Y,  05134AY, 05134JY, 05134KY,  05134ZY, 051407Y, 051409Y,  05140AY, 05140JY, 05140KY,  05140ZY, 051447Y, 051449Y,  05144AY, 05144JY, 05144KY,  05144ZY, 051507Y, 051509Y,  05150AY, 05150JY, 05150KY,  05150ZY, 051547Y, 051549Y,  05154AY, 05154JY, 05154KY,  05154ZY, 051607Y, 051609Y,  05160AY, 05160JY, 05160KY,  05160ZY, 051647Y, 051649Y,  05164AY, 05164JY, 05164KY, 05164ZY, 059Y00Z, 059Y0ZZ,  059Y30Z, 059Y3ZZ, 05C00ZZ,  05C03ZZ, 05C04ZZ, 05C10ZZ,  05C13ZZ, 05C14ZZ, 05C30ZZ,  05C33ZZ, 05C34ZZ, 05C40ZZ,  05C43ZZ, 05C44ZZ, 05C50ZZ,  05C53ZZ, 05C54ZZ, 05C60ZZ,  05C63ZZ, 05C64ZZ, 05C70ZZ,  05C73ZZ, 05C74ZZ, 05C80ZZ,  05C83ZZ, 05C84ZZ, 05C90ZZ,  05C93ZZ, 05C94ZZ, 05CA0ZZ,  05CA3ZZ, 05CA4ZZ, 05CB0ZZ,  05CB3ZZ, 05CB4ZZ, 05CC0ZZ,  05CC3ZZ, 05CC4ZZ, 05CD0ZZ,  05CD3ZZ, 05CD4ZZ, 05CF0ZZ,  05CF3ZZ, 05CF4ZZ, 05CG0ZZ,  05CG3ZZ, 05CG4ZZ, 05CH0ZZ,  05CH3ZZ, 05CH4ZZ, 05CL0ZZ,  05CL4ZZ, 05CM0ZZ, 05CM3ZZ,  05CM4ZZ, 05CN0ZZ, 05CN3ZZ,  05CN4ZZ, 05CP0ZZ, 05CP3ZZ,  05CP4ZZ, 05CQ0ZZ, 05CQ3ZZ,  05CQ4ZZ, 05CR0ZZ, 05CR3ZZ,  05CR4ZZ, 05CS0ZZ, 05CS3ZZ,  05CS4ZZ, 05CT0ZZ, 05CT3ZZ,  05CT4ZZ, 05CV0ZZ, 05CV3ZZ,  05CV4ZZ, 05CY0ZZ, 05CY3ZZ,  05CY4ZZ, 05HY02Z, 05HY32Z,  05HY42Z, 069300Z, 06930ZZ,  069330Z, 06933ZZ, 069340Z,  06934ZZ, 069Y00Z, 069Y0ZZ,  069Y30Z, 069Y3ZZ, 06C00ZZ,  06C03ZZ, 06C04ZZ, 06C10ZZ,  06C13ZZ, 06C14ZZ, 06C20ZZ,  06C23ZZ, 06C24ZZ, 06C30ZZ,  06C33ZZ, 06C34ZZ, 06C40ZZ,  06C43ZZ, 06C44ZZ, 06C50ZZ,  06C53ZZ, 06C54ZZ, 06C60ZZ,  06C63ZZ, 06C64ZZ, 06C70ZZ,  06C73ZZ, 06C74ZZ, 06C80ZZ,  06C83ZZ, 06C84ZZ, 06C90ZZ, 06C93ZZ, 06C94ZZ, 06CB0ZZ,  06CB3ZZ, 06CB4ZZ, 06CC0ZZ,  06CC3ZZ, 06CC4ZZ, 06CD0ZZ,  06CD3ZZ, 06CD4ZZ, 06CF0ZZ,  06CF3ZZ, 06CF4ZZ, 06CG0ZZ,  06CG3ZZ, 06CG4ZZ, 06CH0ZZ,  06CH3ZZ, 06CH4ZZ, 06CJ0ZZ,  06CJ3ZZ, 06CJ4ZZ, 06CM0ZZ,  06CM3ZZ, 06CM4ZZ, 06CN0ZZ,  06CN3ZZ, 06CN4ZZ, 06CP0ZZ,  06CP3ZZ, 06CP4ZZ, 06CQ0ZZ,  06CQ3ZZ, 06CQ4ZZ, 06CR0ZZ,  06CR3ZZ, 06CR4ZZ, 06CS0ZZ,  06CS3ZZ, 06CS4ZZ, 06CT0ZZ,  06CT3ZZ, 06CT4ZZ, 06CV0ZZ,  06CV3ZZ, 06CV4ZZ, 06CY0ZZ,  06CY3ZZ, 06CY4ZZ, 06HY02Z,  06HY32Z, 06HY42Z, 0210098,  0210099, 021009C, 021009F,  021009W, 02100A3, 02100A8,  02100A9, 02100AC, 02100AF,  02100AW, 02100J3, 02100J8,  02100J9, 02100JC, 02100JF,  02100JW, 02100K3, 02100K8,  02100K9, 02100KC, 02100KF,  02100KW, 02100Z3, 02100Z8,  02100Z9, 02100ZC, 02100ZF,  0210493, 0210498, 0210499,  021049C, 021049F, 021049W,  02104A3, 02104A8, 02104A9,  02104AC, 02104AF, 02104AW, 02104J3, 02104J8, 02104J9,  02104JC, 02104JF, 02104JW,  02104K3, 02104K8, 02104K9,  02104KC, 02104KF, 02104KW,  02104Z3, 02104Z8, 02104Z9,  02104ZC, 02104ZF, 0211098,  0211099, 021109C, 021109W,  02110A8, 02110A9, 02110AC,  02110AW, 02110J8, 02110J9,  02110JC, 02110JW, 02110K8,  02110K9, 02110KC, 02110KW,  02110Z8, 02110Z9, 02110ZC,  0211498, 0211499, 021149C,  021149W, 02114A8, 02114A9,  02114AC, 02114AW, 02114J8,  02114J9, 02114JC, 02114JW,  02114K8, 02114K9, 02114KC,  02114KW, 02114Z8, 02114Z9,  02114ZC, 021209C, 021209W,  02120AC, 02120AW, 02120JC,  02120JW, 02120KC, 02120KW,  02120ZC, 021249C, 021249W,  02124AC, 02124AW, 02124JC,  02124JW, 02124KC, 02124KW,  02124ZC, 021309C, 021309W,  02130AC, 02130AW, 02130JC,  02130JW, 02130KC, 02130KW,  02130ZC, 021349C, 021349W,  02134AC, 02134AW, 02134JC,  02134JW, 02134KC, 02134KW,  02134ZC, 021K0Z8, 021K0Z9,  021K0ZC, 021K0ZW, 021K4Z8,  021K4Z9, 021K4ZC, 021K4ZW,  021L0Z8, 021L0Z9, 021L0ZC,  021L4Z8, 021L4Z9, 021L4ZC,  02700ZZ, 02710ZZ, 02720ZZ,  02730ZZ, 02C00ZZ, 02C03ZZ,  02C04ZZ, 02C10ZZ, 02C13ZZ,  02C14ZZ, 02C20ZZ, 02C23ZZ,  02C24ZZ, 02C30ZZ, 02C33ZZ,  02C34ZZ, 3E07017, 3E070PZ, 3E07317, 3E073PZ  Peripheral arterial procedures  021W09B, 021W09D, 021W0AB,  021W0AD, 021W0JB, 021W0JD,  021W0KB, 021W0KD, 021W0ZB, 021W0ZD, 021W49B,  021W49D, 021W4AB, 021W4AD, 021W4JB, 021W4JD,  021W4KB, 021W4KD, 021W4ZB, 021W4ZD, 02CP0ZZ,  02CP3ZZ, 02CP4ZZ, 02CQ0ZZ,  02CQ3ZZ, 02CQ4ZZ, 02CR0ZZ,  02CR3ZZ, 02CR4ZZ, 02CS0ZZ,  02CS3ZZ, 02CS4ZZ, 02CT0ZZ,  02CT3ZZ, 02CT4ZZ, 02CV0ZZ,  02CV3ZZ, 02CV4ZZ, 02CW0ZZ,  02CW3ZZ, 02CW4ZZ, 02HP0DZ,  02HP3DZ, 02HP4DZ, 02HQ0DZ,  02HQ3DZ, 02HQ4DZ, 02HR0DZ,  02HR3DZ, 02HR4DZ, 02HS02Z,  02HS0DZ, 02HS32Z, 02HS3DZ,  02HS42Z, 02HS4DZ, 02HT02Z,  02HT0DZ, 02HT32Z, 02HT3DZ,  02HT42Z, 02HT4DZ, 02HW02Z,  02HW0DZ, 02HW32Z, 02HW3DZ, 02HW42Z, 02HW4DZ, 031H09J, 031H0AJ,  031H0JJ, 031H0KJ, 031H0ZJ,  031J09K, 031J0AK, 031J0JK,  031J0KK, 031J0ZK, 031K09J,  031K0AJ, 031K0JJ, 031K0KJ, 031K0ZJ, 031L09K, 031L0AK, 031L0JK, 031L0KK, 031L0ZK,  031M09J, 031M0AJ, 031M0JJ,  031M0KJ, 031M0ZJ, 031N09K,  031N0AK, 031N0JK, 031N0KK,  031N0ZK, 031S09G, 031S0AG,  031S0JG, 031S0KG, 031S0ZG,  031T09G, 031T0AG, 031T0JG,  031T0KG, 031T0ZG, 039Y0ZZ,  039Y3ZZ, 039Y4ZZ, 03C00ZZ,  03C03ZZ, 03C04ZZ, 03C10ZZ,  03C13ZZ, 03C14ZZ, 03C20ZZ,  03C23ZZ, 03C24ZZ, 03C30ZZ,  03C33ZZ, 03C34ZZ, 03C40ZZ,  03C43ZZ, 03C44ZZ, 03C50ZZ,  03C53ZZ, 03C54ZZ, 03C60ZZ,  03C63ZZ, 03C64ZZ, 03C70ZZ,  03C73ZZ, 03C74ZZ, 03C80ZZ,  03C83ZZ, 03C84ZZ, 03C90ZZ,  03C93ZZ, 03C94ZZ, 03CA0ZZ,  03CA3ZZ, 03CA4ZZ, 03CB0ZZ,  03CB3ZZ, 03CB4ZZ, 03CC0ZZ,  03CC3ZZ, 03CC4ZZ, 03CD0ZZ,  03CD3ZZ, 03CD4ZZ, 03CF0ZZ,  03CF3ZZ, 03CF4ZZ, 03CG0ZZ,  03CG4ZZ, 03CH0ZZ, 03CH4ZZ,  03CJ0ZZ, 03CJ4ZZ, 03CK0ZZ,  03CK4ZZ, 03CL0ZZ, 03CL4ZZ,  03CM0ZZ, 03CM4ZZ, 03CN0ZZ,  03CN4ZZ, 03CP0ZZ, 03CP4ZZ,  03CQ0ZZ, 03CQ4ZZ, 03CR0ZZ,  03CR3ZZ, 03CR4ZZ, 03CS0ZZ,  03CS3ZZ, 03CS4ZZ, 03CT0ZZ,  03CT3ZZ, 03CT4ZZ, 03CU0ZZ,  03CU3ZZ, 03CU4ZZ, 03CV0ZZ,  03CV3ZZ, 03CV4ZZ, 03CY0ZZ,  03CY3ZZ, 03CY4ZZ, 03HY02Z,  03HY32Z, 03HY42Z, 0410090,  0410091, 0410092, 0410093,  0410094, 0410095, 0410096,  0410097, 0410098, 0410099, 041009B, 041009C, 041009D,  041009F, 041009G, 041009H,  041009J, 041009K, 041009Q,  041009R, 04100A0, 04100A1,  04100A2, 04100A3, 04100A4,  04100A5, 04100A6, 04100A7,  04100A8, 04100A9, 04100AB,  04100AC, 04100AD, 04100AF,  04100AG, 04100AH, 04100AJ,  04100AK, 04100AQ, 04100AR,  04100J0, 04100J1, 04100J2,  04100J3, 04100J4, 04100J5,  04100J6, 04100J7, 04100J8,  04100J9, 04100JB, 04100JC,  04100JD, 04100JF, 04100JG,  04100JH, 04100JJ, 04100JK,  04100JQ, 04100JR, 04100K0,  04100K1, 04100K2, 04100K3,  04100K4, 04100K5, 04100K6,  04100K7, 04100K8, 04100K9,  04100KB, 04100KC, 04100KD,  04100KF, 04100KG, 04100KH,  04100KJ, 04100KK, 04100KQ,  04100KR, 04100Z0, 04100Z1,  04100Z2, 04100Z3, 04100Z4,  04100Z5, 04100Z6, 04100Z7,  04100Z8, 04100Z9, 04100ZB,  04100ZC, 04100ZD, 04100ZF,  04100ZG, 04100ZH, 04100ZJ,  04100ZK, 04100ZQ, 04100ZR,  0410490, 0410491, 0410492,  0410493, 0410494, 0410495,  0410496, 0410497, 0410498,  0410499, 041049B, 041049C,  041049D, 041049F, 041049G,  041049H, 041049J, 041049K,  041049Q, 041049R, 04104A0,  04104A1, 04104A2, 04104A3,  04104A4, 04104A5, 04104A6,  04104A7, 04104A8, 04104A9,  04104AB, 04104AC, 04104AD, 04104AF, 04104AG, 04104AH,  04104AJ, 04104AK, 04104AQ,  04104AR, 04104J0, 04104J1,  04104J2, 04104J3, 04104J4,  04104J5, 04104J6, 04104J7,  04104J8, 04104J9, 04104JB,  04104JC, 04104JD, 04104JF,  04104JG, 04104JH, 04104JJ,  04104JK, 04104JQ, 04104JR,  04104K0, 04104K1, 04104K2,  04104K3, 04104K4, 04104K5,  04104K6, 04104K7, 04104K8,  04104K9, 04104KB, 04104KC,  04104KD, 04104KF, 04104KG,  04104KH, 04104KJ, 04104KK,  04104KQ, 04104KR, 04104Z0,  04104Z1, 04104Z2, 04104Z3,  04104Z4, 04104Z5, 04104Z6,  04104Z7, 04104Z8, 04104Z9,  04104ZB, 04104ZC, 04104ZD,  04104ZF, 04104ZG, 04104ZH,  04104ZJ, 04104ZK, 04104ZQ,  04104ZR, 0414093, 0414094,  0414095, 04140A3, 04140A4,  04140A5, 04140J3, 04140J4,  04140J5, 04140K3, 04140K4,  04140K5, 04140Z3, 04140Z4,  04140Z5, 0414493, 0414494,  0414495, 04144A3,04144A4, 04144A5, 04144J3, 04144J4,  04144J5, 04144K3, 04144K4,  04144K5, 04144Z3,04144Z4,  04144Z5, 041C090, 041C091,  041C092, 041C093, 041C094,  041C095, 041C096, 041C097,  041C098, 041C099, 041C09B,  041C09C, 041C09D, 041C09F,  041C09G, 041C09H, 041C09J,  041C09K, 041C09Q, 041C09R,  041C0A0, 041C0A1, 041C0A2,  041C0A3, 041C0A4, 041C0A5, 041C0A6, 041C0A7, 041C0A8,  041C0A9, 041C0AB, 041C0AC,  041C0AD, 041C0AF, 041C0AG,  041C0AH, 041C0AJ, 041C0AK,  041C0AQ, 041C0AR, 041C0J0,  041C0J1, 041C0J2, 041C0J3,  041C0J4, 041C0J5, 041C0J6,  041C0J7, 041C0J8, 041C0J9,  041C0JB, 041C0JC, 041C0JD,  041C0JF, 041C0JG, 041C0JH,  041C0JJ, 041C0JK, 041C0JQ,  041C0JR, 041C0K0, 041C0K1,  041C0K2, 041C0K3, 041C0K4,  041C0K5, 041C0K6, 041C0K7,  041C0K8, 041C0K9, 041C0KB,  041C0KC, 041C0KD, 041C0KF,  041C0KG, 041C0KH, 041C0KJ,  041C0KK, 041C0KQ, 041C0KR,  041C0Z0, 041C0Z1, 041C0Z2,  041C0Z3, 041C0Z4, 041C0Z5,  041C0Z6, 041C0Z7, 041C0Z8,  041C0Z9, 041C0ZB, 041C0ZC,  041C0ZD, 041C0ZF, 041C0ZG,  041C0ZH, 041C0ZJ, 041C0ZK,  041C0ZQ, 041C0ZR, 041C490,  041C491, 041C492, 041C493,  041C494, 041C495, 041C496,  041C497, 041C498, 041C499,  041C49B, 041C49C, 041C49D,  041C49F, 041C49G, 041C49H,  041C49J, 041C49K, 041C49Q,  041C49R, 041C4A0, 041C4A1,  041C4A2, 041C4A3, 041C4A4,  041C4A5, 041C4A6, 041C4A7,  041C4A8, 041C4A9, 041C4AB,  041C4AC, 041C4AD, 041C4AF,  041C4AG, 041C4AH, 041C4AJ,  041C4AK, 041C4AQ, 041C4AR,  041C4J0, 041C4J1, 041C4J2,  041C4J3, 041C4J4, 041C4J5,  041C4J6, 041C4J7, 041C4J8, 041C4J9, 041C4JB, 041C4JC,  041C4JD, 041C4JF, 041C4JG,  041C4JH, 041C4JJ, 041C4JK,  041C4JQ, 041C4JR, 041C4K0,  041C4K1, 041C4K2, 041C4K3,  041C4K4, 041C4K5, 041C4K6,  041C4K7, 041C4K8, 041C4K9,  041C4KB, 041C4KC, 041C4KD,  041C4KF, 041C4KG, 041C4KH,  041C4KJ, 041C4KK, 041C4KQ,  041C4KR, 041C4Z0, 041C4Z1,  041C4Z2, 041C4Z3, 041C4Z4,  041C4Z5, 041C4Z6, 041C4Z7,  041C4Z8, 041C4Z9, 041C4ZB,  041C4ZC, 041C4ZD, 041C4ZF,  041C4ZG, 041C4ZH, 041C4ZJ,  041C4ZK, 041C4ZQ, 041C4ZR,  041D090, 041D091, 041D092,  041D093, 041D094, 041D095,  041D096, 041D097, 041D098,  041D099, 041D09B, 041D09C,  041D09D, 041D09F, 041D09G,  041D09H, 041D09J, 041D09K,  041D09Q, 041D09R, 041D0A0,  041D0A1, 041D0A2, 041D0A3,  041D0A4, 041D0A5, 041D0A6,  041D0A7, 041D0A8, 041D0A9,  041D0AB, 041D0AC, 041D0AD,  041D0AF, 041D0AG, 041D0AH,  041D0AJ, 041D0AK, 041D0AQ,  041D0AR, 041D0J0, 041D0J1,  041D0J2, 041D0J3, 041D0J4,  041D0J5, 041D0J6, 041D0J7,  041D0J8, 041D0J9, 041D0JB,  041D0JC, 041D0JD, 041D0JF,  041D0JG, 041D0JH, 041D0JJ,  041D0JK, 041D0JQ, 041D0JR,  041D0K0, 041D0K1, 041D0K2,  041D0K3, 041D0K4, 041D0K5,  041D0K6, 041D0K7, 041D0K8,  041D0K9, 041D0KB, 041D0KC, 041D0KD, 041D0KF, 041D0KG,  041D0KH, 041D0KJ, 041D0KK,  041D0KQ, 041D0KR, 041D0Z0,  041D0Z1, 041D0Z2, 041D0Z3,  041D0Z4, 041D0Z5, 041D0Z6,  041D0Z7, 041D0Z8, 041D0Z9,  041D0ZB, 041D0ZC, 041D0ZD,  041D0ZF, 041D0ZG, 041D0ZH,  041D0ZJ, 041D0ZK, 041D0ZQ,  041D0ZR, 041D490, 041D491,  041D492, 041D493, 041D494,  041D495, 041D496, 041D497,  041D498, 041D499, 041D49B,  041D49C, 041D49D, 041D49F,  041D49G, 041D49H, 041D49J,  041D49K, 041D49Q, 041D49R,  041D4A0, 041D4A1, 041D4A2,  041D4A3, 041D4A4, 041D4A5,  041D4A6, 041D4A7, 041D4A8,  041D4A9, 041D4AB, 041D4AC,  041D4AD, 041D4AF, 041D4AG,  041D4AH, 041D4AJ, 041D4AK,  041D4AQ, 041D4AR, 041D4J0,  041D4J1, 041D4J2, 041D4J3,  041D4J4, 041D4J5, 041D4J6,  041D4J7, 041D4J8, 041D4J9,  041D4JB, 041D4JC, 041D4JD,  041D4JF, 041D4JG, 041D4JH,  041D4JJ, 041D4JK, 041D4JQ,  041D4JR, 041D4K0, 041D4K1,  041D4K2, 041D4K3, 041D4K4,  041D4K5, 041D4K6, 041D4K7,  041D4K8, 041D4K9, 041D4KB,  041D4KC, 041D4KD, 041D4KF,  041D4KG, 041D4KH, 041D4KJ,  041D4KK, 041D4KQ, 041D4KR,  041D4Z0, 041D4Z1, 041D4Z2,  041D4Z3, 041D4Z4, 041D4Z5,  041D4Z6, 041D4Z7, 041D4Z8,  041D4Z9, 041D4ZB, 041D4ZC,  041D4ZD, 041D4ZF, 041D4ZG, 041D4ZH, 041D4ZJ, 041D4ZK, 041D4ZQ, 041D4ZR, 041E099,  041E09B, 041E09C, 041E09D,  041E09F, 041E09G, 041E09H,  041E09J, 041E09K, 041E09P,  041E09Q, 041E0A9, 041E0AB,  041E0AC, 041E0AD, 041E0AF,  041E0AG, 041E0AH, 041E0AJ,  041E0AK, 041E0AP, 041E0AQ,  041E0J9, 041E0JB, 041E0JC,  041E0JD, 041E0JF, 041E0JG,  041E0JH, 041E0JJ, 041E0JK,  041E0JP, 041E0JQ, 041E0K9,  041E0KB, 041E0KC, 041E0KD,  041E0KF, 041E0KG, 041E0KH,  041E0KJ, 041E0KK, 041E0KP,  041E0KQ, 041E0Z9, 041E0ZB,  041E0ZC, 041E0ZD, 041E0ZF,  041E0ZG, 041E0ZH, 041E0ZJ,  041E0ZK, 041E0ZP, 041E0ZQ,  041E499, 041E49B, 041E49C,  041E49D, 041E49F, 041E49G,  041E49H, 041E49J, 041E49K,  041E49P, 041E49Q, 041E4A9,  041E4AB, 041E4AC, 041E4AD,  041E4AF, 041E4AG, 041E4AH,  041E4AJ, 041E4AK, 041E4AP,  041E4AQ, 041E4J9, 041E4JB,  041E4JC, 041E4JD, 041E4JF,  041E4JG, 041E4JH, 041E4JJ,  041E4JK, 041E4JP, 041E4JQ,  041E4K9, 041E4KB, 041E4KC,  041E4KD, 041E4KF, 041E4KG,  041E4KH, 041E4KJ, 041E4KK,  041E4KP, 041E4KQ, 041E4Z9,  041E4ZB, 041E4ZC, 041E4ZD,  041E4ZF, 041E4ZG, 041E4ZH,  041E4ZJ, 041E4ZK, 041E4ZP,  041E4ZQ, 041F099, 041F09B,  041F09C, 041F09D, 041F09F,  041F09G, 041F09H, 041F09J, 041F09K, 041F09P, 041F09Q,  041F0A9, 041F0AB, 041F0AC,  041F0AD, 041F0AF, 041F0AG,  041F0AH, 041F0AJ, 041F0AK,  041F0AP, 041F0AQ, 041F0J9,  041F0JB, 041F0JC, 041F0JD,  041F0JF, 041F0JG, 041F0JH,  041F0JJ, 041F0JK, 041F0JP,  041F0JQ, 041F0K9, 041F0KB,  041F0KC, 041F0KD, 041F0KF,  041F0KG, 041F0KH, 041F0KJ,  041F0KK, 041F0KP, 041F0KQ,  041F0Z9, 041F0ZB, 041F0ZC,  041F0ZD, 041F0ZF, 041F0ZG,  041F0ZH, 041F0ZJ, 041F0ZK,  041F0ZP, 041F0ZQ, 041F499,  041F49B, 041F49C, 041F49D,  041F49F, 041F49G, 041F49H,  041F49J, 041F49K, 041F49P,  041F49Q, 041F4A9, 041F4AB,  041F4AC, 041F4AD, 041F4AF,  041F4AG, 041F4AH, 041F4AJ,  041F4AK, 041F4AP, 041F4AQ,  041F4J9, 041F4JB, 041F4JC,  041F4JD, 041F4JF, 041F4JG,  041F4JH, 041F4JJ, 041F4JK,  041F4JP, 041F4JQ, 041F4K9,  041F4KB, 041F4KC, 041F4KD,  041F4KF, 041F4KG, 041F4KH,  041F4KJ, 041F4KK, 041F4KP,  041F4KQ, 041F4Z9, 041F4ZB,  041F4ZC, 041F4ZD, 041F4ZF,  041F4ZG, 041F4ZH, 041F4ZJ,  041F4ZK, 041F4ZP, 041F4ZQ,  041H099, 041H09B, 041H09C,  041H09D, 041H09F, 041H09G,  041H09H, 041H09J, 041H09K,  041H09P, 041H09Q, 041H0A9,  041H0AB, 041H0AC, 041H0AD,  041H0AF, 041H0AG, 041H0AH,  041H0AJ, 041H0AK, 041H0AP, 041H0AQ, 041H0J9, 041H0JB, 041H0JC, 041H0JD, 041H0JF,  041H0JG, 041H0JH, 041H0JJ,  041H0JK, 041H0JP, 041H0JQ,  041H0K9, 041H0KB, 041H0KC,  041H0KD, 041H0KF, 041H0KG,  041H0KH, 041H0KJ, 041H0KK,  041H0KP, 041H0KQ, 041H0Z9,  041H0ZB, 041H0ZC, 041H0ZD,  041H0ZF, 041H0ZG, 041H0ZH,  041H0ZJ, 041H0ZK, 041H0ZP,  041H0ZQ, 041H499, 041H49B,  041H49C, 041H49D, 041H49F,  041H49G, 041H49H, 041H49J,  041H49K, 041H49P, 041H49Q, 041H4A9, 041H4AB, 041H4AC,  041H4AD, 041H4AF, 041H4AG,  041H4AH, 041H4AJ, 041H4AK,  041H4AP, 041H4AQ, 041H4J9,  041H4JB, 041H4JC, 041H4JD,  041H4JF, 041H4JG, 041H4JH,  041H4JJ, 041H4JK, 041H4JP,  041H4JQ, 041H4K9, 041H4KB,  041H4KC, 041H4KD, 041H4KF,  041H4KG, 041H4KH, 041H4KJ,  041H4KK, 041H4KP, 041H4KQ,  041H4Z9, 041H4ZB, 041H4ZC,  041H4ZD, 041H4ZF, 041H4ZG,  041H4ZH, 041H4ZJ, 041H4ZK,  041H4ZP, 041H4ZQ, 041J099,  041J09B, 041J09C, 041J09D,  041J09F, 041J09G, 041J09H,  041J09J, 041J09K, 041J09P,  041J09Q, 041J0A9, 041J0AB,  041J0AC, 041J0AD, 041J0AF,  041J0AG, 041J0AH, 041J0AJ,  041J0AK, 041J0AP, 041J0AQ,  041J0J9, 041J0JB, 041J0JC,  041J0JD, 041J0JF, 041J0JG,  041J0JH, 041J0JJ, 041J0JK,  041J0JP, 041J0JQ, 041J0K9, 041J0KB, 041J0KC, 041J0KD, 041J0KF, 041J0KG, 041J0KH,  041J0KJ, 041J0KK, 041J0KP,  041J0KQ, 041J0Z9, 041J0ZB,  041J0ZC, 041J0ZD, 041J0ZF,  041J0ZG, 041J0ZH, 041J0ZJ,  041J0ZK, 041J0ZP, 041J0ZQ,  041J499, 041J49B, 041J49C,  041J49D, 041J49F, 041J49G,  041J49H, 041J49J, 041J49K,  041J49P, 041J49Q, 041J4A9,  041J4AB, 041J4AC, 041J4AD,  041J4AF, 041J4AG, 041J4AH,  041J4AJ, 041J4AK, 041J4AP,  041J4AQ, 041J4J9, 041J4JB, 041J4JC, 041J4JD, 041J4JF,  041J4JG, 041J4JH, 041J4JJ,  041J4JK, 041J4JP, 041J4JQ,  041J4K9, 041J4KB, 041J4KC,  041J4KD, 041J4KF, 041J4KG,  041J4KH, 041J4KJ, 041J4KK,  041J4KP, 041J4KQ, 041J4Z9,  041J4ZB, 041J4ZC, 041J4ZD,  041J4ZF, 041J4ZG, 041J4ZH,  041J4ZJ, 041J4ZK, 041J4ZP,  041J4ZQ, 049Y0ZZ, 049Y3ZZ,  049Y4ZZ, 04C00ZZ, 04C03ZZ,  04C04ZZ, 04C10ZZ, 04C13ZZ,  04C14ZZ, 04C20ZZ, 04C23ZZ,  04C24ZZ, 04C30ZZ, 04C33ZZ,  04C34ZZ, 04C40ZZ, 04C43ZZ,  04C44ZZ, 04C50ZZ, 04C53ZZ,  04C54ZZ, 04C60ZZ, 04C63ZZ,  04C64ZZ, 04C70ZZ, 04C73ZZ,  04C74ZZ, 04C80ZZ, 04C83ZZ,  04C84ZZ, 04C90ZZ, 04C93ZZ,  04C94ZZ, 04CA0ZZ, 04CA3ZZ,  04CA4ZZ, 04CB0ZZ, 04CB3ZZ,  04CB4ZZ, 04CC0ZZ, 04CC3ZZ,  04CC4ZZ, 04CD0ZZ, 04CD3ZZ,  04CD4ZZ, 04CE0ZZ, 04CE3ZZ, 04CE4ZZ, 04CF0ZZ, 04CF3ZZ, 04CF4ZZ, 04CH0ZZ, 04CH3ZZ,  04CH4ZZ, 04CJ0ZZ, 04CJ3ZZ,  04CJ4ZZ, 04CK0ZZ, 04CK3ZZ,  04CK4ZZ, 04CL0ZZ, 04CL3ZZ,  04CL4ZZ, 04CM0ZZ, 04CM3ZZ,  04CM4ZZ, 04CN0ZZ, 04CN3ZZ,  04CN4ZZ, 04CP0ZZ, 04CP3ZZ,  04CP4ZZ, 04CQ0ZZ, 04CQ3ZZ,  04CQ4ZZ, 04CR0ZZ, 04CR3ZZ,  04CR4ZZ, 04CS0ZZ, 04CS3ZZ,  04CS4ZZ, 04CT0ZZ, 04CT3ZZ,  04CT4ZZ, 04CU0ZZ, 04CU3ZZ,  04CU4ZZ, 04CV0ZZ, 04CV3ZZ,  04CV4ZZ, 04CW0ZZ, 04CW3ZZ,  04CW4ZZ, 04CY0ZZ, 04CY3ZZ,  04CY4ZZ, 04HY02Z, 04HY32Z,  04HY42Z, 051007Y, 051009Y,  05100AY, 05100JY, 05100KY,  05100ZY, 051047Y, 051049Y,  05104AY, 05104JY, 05104KY,  05104ZY, 051107Y, 051109Y,  05110AY, 05110JY, 05110KY,  05110ZY, 051147Y, 051149Y,  05114AY, 05114JY, 05114KY,  05114ZY, 051307Y, 051309Y,  05130AY, 05130JY, 05130KY,  05130ZY, 051347Y, 051349Y,  05134AY, 05134JY, 05134KY,  05134ZY, 051407Y, 051409Y,  05140AY, 05140JY, 05140KY,  05140ZY, 051447Y, 051449Y,  05144AY, 05144JY, 05144KY,  05144ZY, 051507Y, 051509Y,  05150AY, 05150JY,05150KY,  05150ZY, 051547Y, 051549Y,  05154AY, 05154JY, 05154KY,  05154ZY, 051607Y, 051609Y,  05160AY, 05160JY, 05160KY,  05160ZY, 051647Y, 051649Y,  05164AY, 05164JY, 05164KY, 05164ZY, 059Y00Z, 059Y0ZZ, 059Y30Z, 059Y3ZZ, 05C00ZZ,  05C03ZZ, 05C04ZZ, 05C10ZZ,  05C13ZZ, 05C14ZZ, 05C30ZZ,  05C33ZZ, 05C34ZZ, 05C40ZZ,  05C43ZZ, 05C44ZZ, 05C50ZZ,  05C53ZZ, 05C54ZZ, 05C60ZZ,  05C63ZZ, 05C64ZZ, 05C70ZZ,  05C73ZZ, 05C74ZZ, 05C80ZZ,  05C83ZZ, 05C84ZZ, 05C90ZZ,  05C93ZZ, 05C94ZZ, 05CA0ZZ,  05CA3ZZ, 05CA4ZZ, 05CB0ZZ,  05CB3ZZ, 05CB4ZZ, 05CC0ZZ,  05CC3ZZ, 05CC4ZZ, 05CD0ZZ,  05CD3ZZ, 05CD4ZZ, 05CF0ZZ,  05CF3ZZ, 05CF4ZZ, 05CG0ZZ,  05CG3ZZ, 05CG4ZZ, 05CH0ZZ,  05CH3ZZ, 05CH4ZZ, 05CL0ZZ,  05CL4ZZ, 05CM0ZZ, 05CM3ZZ,  05CM4ZZ, 05CN0ZZ, 05CN3ZZ,  05CN4ZZ, 05CP0ZZ, 05CP3ZZ,  05CP4ZZ, 05CQ0ZZ, 05CQ3ZZ,  05CQ4ZZ, 05CR0ZZ, 05CR3ZZ,  05CR4ZZ, 05CS0ZZ, 05CS3ZZ,  05CS4ZZ, 05CT0ZZ, 05CT3ZZ,  05CT4ZZ, 05CV0ZZ, 05CV3ZZ,  05CV4ZZ, 05CY0ZZ, 05CY3ZZ,  05CY4ZZ, 05HY02Z, 05HY32Z,  05HY42Z, 069300Z, 06930ZZ,  069330Z, 06933ZZ, 069340Z,  06934ZZ, 069Y00Z, 069Y0ZZ,  069Y30Z, 069Y3ZZ, 06C00ZZ,  06C03ZZ, 06C04ZZ, 06C10ZZ,  06C13ZZ, 06C14ZZ, 06C20ZZ,  06C23ZZ, 06C24ZZ, 06C30ZZ,  06C33ZZ, 06C34ZZ, 06C40ZZ,  06C43ZZ, 06C44ZZ, 06C50ZZ,  06C53ZZ, 06C54ZZ, 06C60ZZ,  06C63ZZ, 06C64ZZ, 06C70ZZ,  06C73ZZ, 06C74ZZ, 06C80ZZ,  06C83ZZ, 06C84ZZ, 06C90ZZ, 06C93ZZ, 06C94ZZ, 06CB0ZZ,  06CB3ZZ, 06CB4ZZ, 06CC0ZZ,  06CC3ZZ, 06CC4ZZ, 06CD0ZZ,  06CD3ZZ, 06CD4ZZ, 06CF0ZZ,  06CF3ZZ, 06CF4ZZ, 06CG0ZZ,  06CG3ZZ, 06CG4ZZ, 06CH0ZZ,  06CH3ZZ, 06CH4ZZ, 06CJ0ZZ,  06CJ3ZZ, 06CJ4ZZ, 06CM0ZZ,  06CM3ZZ, 06CM4ZZ, 06CN0ZZ,  06CN3ZZ, 06CN4ZZ, 06CP0ZZ, 06CP3ZZ, 06CP4ZZ, 06CQ0ZZ,  06CQ3ZZ, 06CQ4ZZ, 06CR0ZZ, 06CR3ZZ, 06CR4ZZ, 06CS0ZZ,  06CS3ZZ, 06CS4ZZ, 06CT0ZZ,  06CT3ZZ, 06CT4ZZ, 06CV0ZZ,  06CV3ZZ, 06CV4ZZ, 06CY0ZZ,  06CY3ZZ, 06CY4ZZ, 06HY02Z,  06HY32Z, 06HY42Z |

Supplemental Table 2. Sex-TBI Interaction Analysis Results

| Variable | HR | 95% CI | P value |
| --- | --- | --- | --- |
| TBI Group Main Effect |  |  |  |
| Mild | 1.55 | 1.53-1.57 | <0.001 |
| Moderate/Severe | 2.64 | 2.61-2.67 | <0.001 |
| Penetrating | 4.21 | 4.16-4.26 | <0.001 |
| Sex Main Effect |  |  |  |
| Female vs. Male | 0.59 | 0.58-0.61 | <0.001 |
| Sex * TBI Interaction |  |  |  |
| Mild for Female | 1.45 | 1.40-1.50 | <0.001 |
| Moderate/Severe for Female | 1.44 | 1.40-1.49 | <0.001 |
| Penetrating for Female | 1.62 | 1.56-1.67 | <0.001 |

Supplemental Table 3. Standardized Mean Differences with Inverse Propensity Score Weighting

|  | Mild TBI vs no TBI | | Moderate/Severe TBI vs no TBI | | Penetrating TBI vs no TBI | |
| --- | --- | --- | --- | --- | --- | --- |
|  | Not Weighted | Weighted | Not Weighted | Weighted | Not Weighted | Weighted |
| Birth Year | 0.1923 | -0.009 | 0.1362 | -0.0151 | 0.1067 | -0.0054 |
| Sex | 0.1971 | -0.0741 | 0.2876 | -0.0752 | 0.2957 | -0.0594 |
| Age 17-24 | 0.1713 | 0.1697 | 0.0117 | 0.0903 | 0.0173 | 0.0461 |
| Age 25-34 | 0.0586 | -0.1046 | 0.1391 | -0.0625 | 0.0031 | -0.1078 |
| Age 35-44 | -0.0907 | -0.053 | -0.0285 | -0.0274 | 0.0719 | 0.0153 |
| Age 45-54 | -0.1936 | -0.0388 | -0.1573 | -0.0326 | -0.0908 | 0.0209 |
| Age 55-64 | -0.1184 | 0.0115 | -0.1021 | 0.0354 | -0.0724 | 0.0521 |
| Age 65+ | -0.039 | 0.0321 | -0.0324 | 0.0575 | 0.008 | 0.1115 |
| Asian/Pacific Islander | 0.0835 | -0.0217 | 0.0869 | -0.0023 | 0.084 | 0.0078 |
| Hispanic Black | -0.0116 | 0.002 | -0.0147 | 0.0029 | -0.0176 | 0.0084 |
| Non-Hispanic Black | -0.0952 | 0.014 | -0.1141 | -0.0091 | -0.105 | 0.0049 |
| Hispanic | 0.0059 | -0.0039 | -0.0076 | 0.0003 | 0.0081 | -0.0003 |
| Native American | 0.0283 | -0.0004 | 0.0293 | 0.0036 | 0.0193 | -0.007 |
| Unknown Race | -0.0742 | 0.0152 | -0.1146 | 0.0467 | -0.0692 | 0.0044 |
| Non-Hispanic White | 0.0376 | -0.0024 | 0.066 | -0.007 | 0.0455 | -0.0082 |
| College Graduate | -0.1602 | 0.0202 | -0.1785 | 0.0282 | -0.0884 | 0.0002 |
| Graduate School | -0.1891 | 0.0222 | -0.188 | 0.0325 | -0.1326 | 0.0395 |
| Less than High School | 0.0113 | -0.0033 | 0.0201 | 0.0011 | -0.0046 | -0.0013 |
| Some College | -0.0616 | 0.0033 | -0.0628 | 0.0127 | 0.0067 | -0.0013 |
| Unknown Education | -0.0649 | 0.0088 | -0.0964 | 0.0273 | -0.0604 | 0.0354 |
| High School | 0.2505 | -0.0288 | 0.2656 | -0.0519 | 0.1323 | -0.0282 |
| Air Force | -0.2949 | 0.0375 | -0.3451 | 0.0586 | -0.342 | 0.068 |
| Marines | 0.0974 | -0.0207 | 0.1358 | -0.0329 | 0.131 | -0.054 |
| Navy/Coast Guard | -0.2422 | 0.0287 | -0.2596 | 0.025 | -0.256 | 0.0025 |
| Other Branch | -0.0251 | -0.0003 | -0.0211 | -0.0008 | -0.0131 | 0.0048 |
| Army | 0.3311 | -0.0389 | 0.3439 | -0.0448 | 0.3431 | -0.0204 |
| Guard | 0.0637 | 0.0188 | 0.0475 | 0.041 | -0.0289 | 0.1072 |
| Reserve | -0.0764 | -0.011 | -0.1646 | -0.0031 | -0.4147 | -0.0227 |
| Active | 0.033 | -0.0012 | 0.1269 | -0.0232 | 0.4043 | -0.0491 |
| Enlisted | 0.2557 | -0.0349 | 0.2679 | -0.0506 | 0.1628 | -0.0326 |
| Officer | -0.2512 | 0.0369 | -0.269 | 0.0519 | -0.1845 | 0.0318 |
| Warrant | -0.0463 | -0.0031 | -0.0322 | 0.0016 | 0.0328 | 0.0061 |
| -Combat or Deploy | -0.2927 | 0.0715 | -0.3263 | 0.0824 | -0.2193 | 0.0758 |
| -Combat/+Deploy | -0.0498 | 0.0014 | -0.0763 | 0.0006 | -0.0875 | 0.0168 |
| +Combat/-Deploy | -0.1441 | 0.0171 | -0.1321 | 0.0233 | -0.1387 | 0.0277 |
| +Combat/+Deploy | 0.3483 | -0.0739 | 0.3857 | -0.0865 | 0.2981 | -0.0899 |
| Smoking History | 0.3568 | -0.0205 | 0.4274 | -0.0168 | 0.3857 | -0.0051 |
| Substance Use Disorder | 0.1464 | -0.0021 | 0.2425 | -0.0107 | 0.1893 | 0.0144 |
| Obesity | -0.0019 | 0.0114 | 0.0403 | 0.0051 | 0.0103 | 0.0205 |
| Depression | 0.2977 | 0.0229 | 0.4581 | 0.0196 | 0.2792 | 0.0334 |
| Anxiety | 0.2773 | 0.0142 | 0.4441 | 0.0041 | 0.3428 | 0.0195 |
| Insomnia | 0.289 | 0.0029 | 0.4579 | -0.0107 | 0.4457 | 0.0109 |
| PTSD | 0.5489 | -0.0091 | 0.7754 | -0.0096 | 0.5096 | 0.0187 |
| Hyperlipidemia | -0.0942 | 0.0218 | -0.0277 | 0.0194 | -0.0012 | 0.0371 |
| Hypertension | -0.0338 | 0.0149 | 0.0435 | 0.0271 | 0.0457 | 0.0535 |
| Kidney Disease | -0.0154 | 0.0042 | 0.0056 | 0.0129 | 0.0053 | 0.0146 |
| Diabetes | -0.05 | 0.0182 | -0.0139 | 0.0166 | -0.0043 | 0.038 |
| OSA | 0.0396 | 0.009 | 0.1619 | -0.01 | 0.1683 | 0.0095 |

Supplemental Figure 1. Cumulative incidence functions for the composite of CVD outcomes stratified by sex and traumatic brain injury.


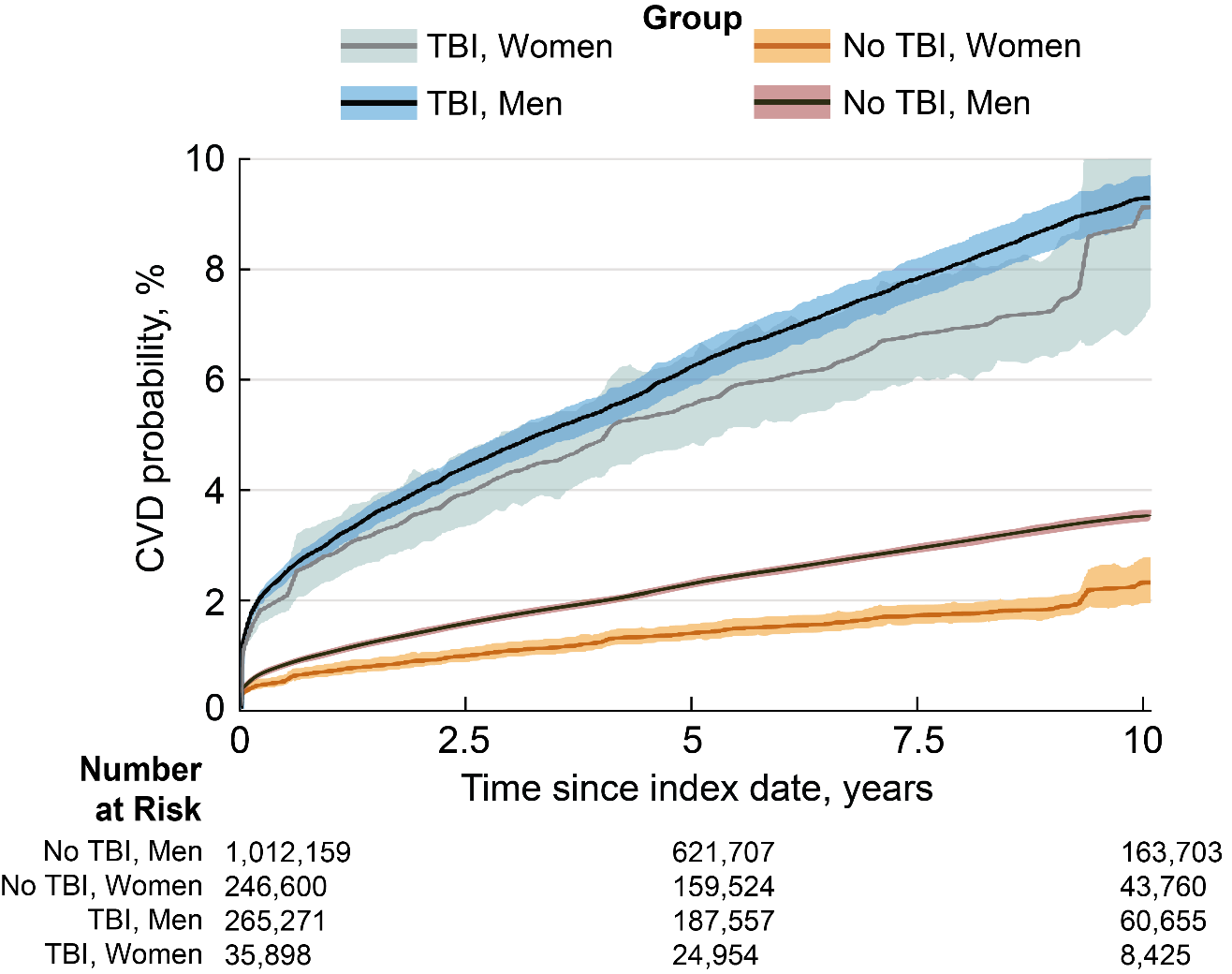


Supplemental Figure 2. Bivariate and Multivariable Competing Risk Models for the Outcome of Cardiovascular Disease


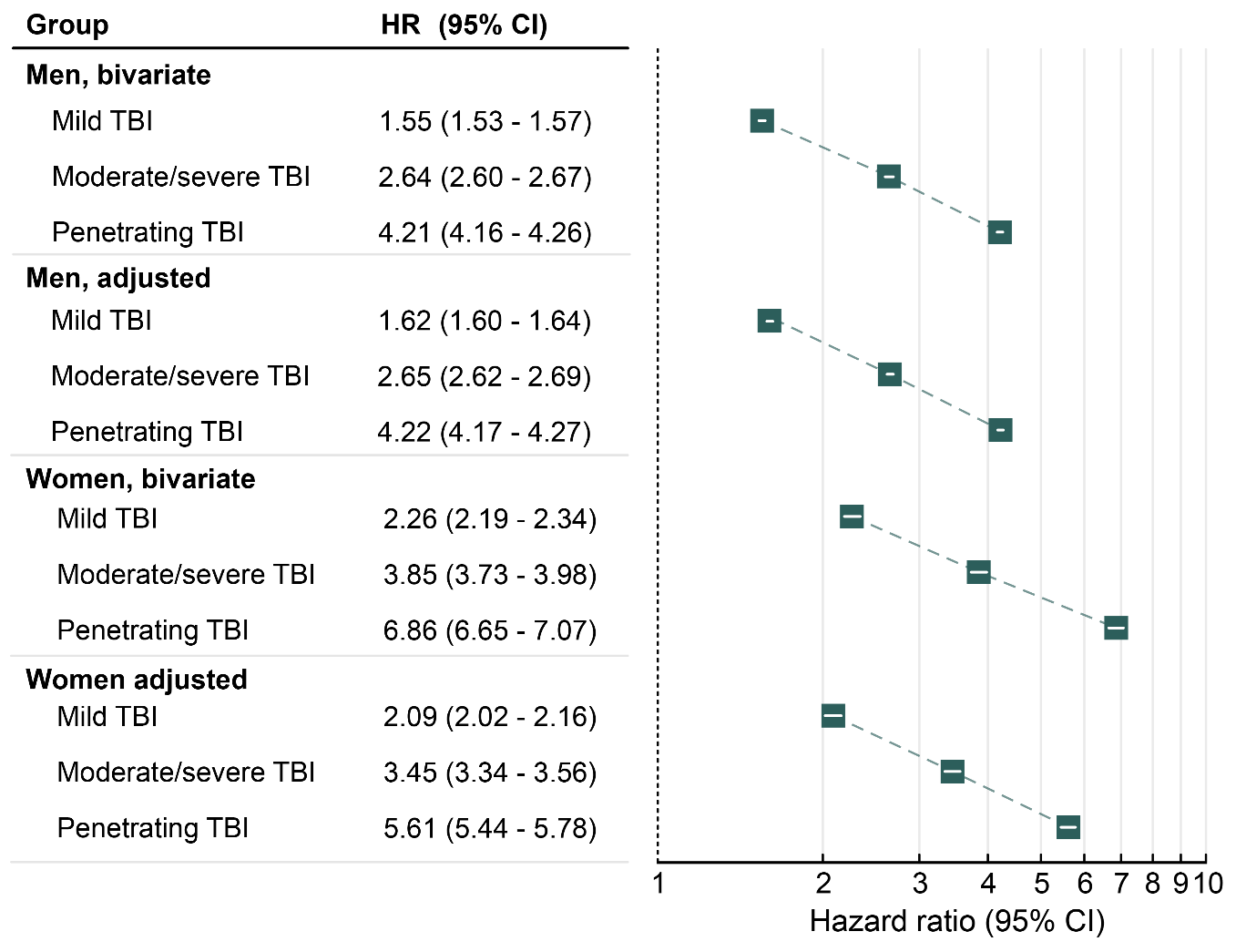

Supplement: Supplemental Tables 1-3 and Supplemental Figures 1 and 2 [file mmc1.docx]
